# S2P intramembrane protease RseP degrades small membrane proteins and suppresses the cytotoxicity of intrinsic toxin HokB

**DOI:** 10.1128/mbio.01086-23

**Published:** 2023-07-06

**Authors:** Tatsuhiko Yokoyama, Yutaro Yamagata, Saisei Honna, Shinya Mizuno, Shizuka Katagiri, Rika Oi, Terukazu Nogi, Yohei Hizukuri, Yoshinori Akiyama

**Affiliations:** 1 Institute for Life and Medical Sciences, Kyoto University, Sakyo-ku, Kyoto, Japan; 2 Graduate School of Medical Life Science, Yokohama City University, Tsurumi-ku, Yokohama, Japan; National Institute of Child Health and Human Development, Bethesda, Maryland, USA

**Keywords:** regulated intramembrane proteolysis, membrane protease, extracytoplasmic stress response, proteostasis, zinc metallopeptidase

## Abstract

The site2*-*protease (S2P) family of intramembrane proteases (IMPs) is conserved in all kingdoms of life and cleaves transmembrane proteins within the membrane to regulate and maintain various cellular activities. RseP, an *Escherichia coli* S2P peptidase, is involved in the regulation of gene expression through the regulated cleavage of the two target membrane proteins (RseA and FecR) and in membrane quality control through the proteolytic elimination of remnant signal peptides. RseP is expected to have additional substrates and to be involved in other cellular processes. Recent studies have shown that cells express small membrane proteins (SMPs; single-spanning membrane proteins of approximately 50–100 amino acid residues) with crucial cellular functions. However, little is known about their metabolism, which affects their functions. This study investigated the possible RseP-catalyzed cleavage of *E. coli* SMPs based on the apparent similarity of the sizes and structures of SMPs to those of remnant signal peptides. We screened SMPs cleaved by RseP *in vivo* and *in vitro* and identified 14 SMPs, including HokB, an endogenous toxin that induces persister formation, as potential substrates. We demonstrated that RseP suppresses the cytotoxicity and biological functions of HokB. The identification of several SMPs as novel potential substrates of RseP provides a clue to a comprehensive understanding of the cellular roles of RseP and other S2P peptidases and highlights a novel aspect of the regulation of SMPs.

**IMPORTANCE:**

Membrane proteins play an important role in cell activity and survival. Thus, understanding their dynamics, including proteolytic degradation, is crucial. *E. coli* RseP, an S2P family intramembrane protease, cleaves membrane proteins to regulate gene expression in response to environmental changes and to maintain membrane quality. To identify novel substrates of RseP, we screened small membrane proteins (SMPs), a group of proteins that have recently been shown to have diverse cellular functions, and identified 14 potential substrates. We also showed that RseP suppresses the cytotoxicity of the intrinsic toxin, HokB, an SMP that has been reported to induce persister cell formation, by degrading it. These findings provide new insights into the cellular roles of S2P peptidases and the functional regulation of SMPs.

## INTRODUCTION

Increasing evidence has demonstrated that intramembrane proteases (IMPs), a category of membrane proteases that cleaves substrate proteins within the membrane, play critical roles in the regulation and maintenance of diverse cellular activities in prokaryotic and eukaryotic cells. IMPs are classified into four families: site2*-*protease (S2P), rhomboid peptidase, presenilin/signal peptide peptidase, and Rce1 (1 - 4). These proteases share a common structural feature: their protease-active sites reside within the membrane plane (5). While members of the first three classes of IMPs cleave transmembrane proteins, Rce1 cleaves prenylated non-transmembrane proteins.

S2P peptidases control various cellular processes, including stress responses, lipid metabolism, membrane quality control, and bacterial pathogenesis and spore formation through the regulated cleavage of substrate membrane proteins (2, 3, 6, 7). *Escherichia coli* RseP, one of the most well-studied S2P peptidases, was first demonstrated to act in the stress-dependent regulation of the σ^E^ extracytoplasmic stress response by cleaving RseA, which binds σ^E^ in its cytoplasmic domain and holds it inactive (8 - 10). Stress signals, such as the accumulation of misfolded outer membrane proteins (OMPs), activate DegS, a membrane protease with periplasmic active sites, to cleave the periplasmic region of RseA, triggering the subsequent RseP-catalyzed intramembrane cleavage of the DegS-processed form of RseA, resulting in the release and the final activation of σ^E^ to regulate the expression of stress-responsive genes (3, 11). Similarly, we have recently shown that RseP regulates the transcription of genes involved in iron transport through intramembrane cleavage of FecR (12). In addition, RseP contributes to the maintenance of the cytoplasmic membrane by degrading signal peptides that are generated during the membrane translocation of secretory proteins and left behind in the membrane (13).

RseP cleaves the transmembrane regions of RseA and FecR after they undergo prior periplasmic cleavage(s). RseP-catalyzed cleavage of signal peptides also requires their proteolytic detachment from the mature region of secretory proteins. The two tandemly arranged periplasmic PDZ domains have been suggested to function as a size-exclusion filter that prevents the access of substrates with a large periplasmic region to the membrane-embedded protease domain of RseP (14 - 16), which was supported by the recent structural elucidation of RseP and its closely related bacterial homolog (17). Structural and biochemical studies have also proposed that the dynamic conformational changes of the peripheral and intramembrane domains of RseP act as a gate to modulate the entry of the transmembrane segment (TM) of periplasmically processed substrates into the hydrophilic active site compartment formed in the membrane (17, 18). A membrane-reentrant β-sheet structure (MRE β-sheet) located adjacent to the active site that directly binds and extends a substrate TM for proteolysis would also contribute to substrate discrimination by RseP (17, 19, 20).

The observation that RseP can cleave many transmembrane sequences with no apparent amino acid sequence similarity or conserved motifs raises the possibility that additional, unidentified RseP substrates exist. A systematic mass spectroscopy-based proteomic analysis based on this assumption led to the discovery of FecR as a novel substrate of RseP (12). However, this approach failed to identify any other novel or known substrates, including signal peptides, presumably because of the difficulty in analyzing hydrophobic, relatively small membrane proteins. Thus, the identification of RseP substrates is likely to be incomplete. Consequently, the cellular functions of RseP remain unclear.

Recent genomic and proteomic analyses have revealed that cells express a group of small proteins smaller than about 50 amino acids long in prokaryotes and 100 amino acids long in eukaryotes, which were overlooked in the early days of the genomic studies due to the difficulties in annotation and characterization (21). Approximately one-third of the identified small proteins are predicted to contain a transmembrane helix (22 - 24) and are called small membrane proteins (SMPs). Although the number of SMPs expressed and the physiological roles they play are not fully understood, an increasing number of SMPs has been found to be expressed in prokaryotic and eukaryotic cells. For instance, *E. coli* SMPs are involved in various cellular activities, including signal transduction, membrane protein degradation, cell division, respiration, and transport across the membrane (23, 24).

However, knowledge of the regulatory mechanisms of SMP functions is rather limited. Modification and ligand binding in extramembrane regions are well-known mechanisms that qualitatively modulate the functions of membrane proteins. However, in the case of SMPs, such regulation appears difficult because they have only small extramembrane domains. As for quantitative control, while some SMPs are reportedly regulated at the biogenesis step (23), little is known about the control of their stability by proteolytic degradation, although this could be another important mechanism to alter the cellular abundance and regulate the function of SMPs. While two membrane proteases, FtsH and HtpX, have been suggested to play a major role in the proteolytic quality control of membrane proteins in *E. coli*, they would have difficulty in the degradation of membrane proteins with small extramembrane regions, as their active sites are located in the cytoplasmically exposed domains. FtsH has been shown to degrade membrane proteins with a certain length of N- or C-terminal cytoplasmic tail (25, 26). In contrast, RseP can catalyze the intramembrane proteolysis of signal peptides, which in general have very small, or even almost no, extramembrane regions. It is thus reasonable to assume that some SMPs, structurally similar to signal peptides, could also become substrates of RseP. In support of this idea, we have previously found that YqfG, an SMP, is cleaved by RseP (20).

In this study, we screened SMPs and identified several potential substrates of RseP that were cleaved by RseP *in vivo* and/or *in vitro*. We demonstrated that RseP cleaves HokB, an endogenous toxin (27) that induces the formation of persisters (a small fraction of an isogenic population that transiently exhibits tolerance to antibiotics) (28 - 31) and suppresses several biological functions of HokB. Our results highlight new aspects of the cellular roles of S2P peptidases and the functional regulation of SMPs by intramembrane proteolysis.

## RESULTS

### *In vivo* screening for small membrane proteins (SMPs) cleaved by RseP

**Fig 1:**
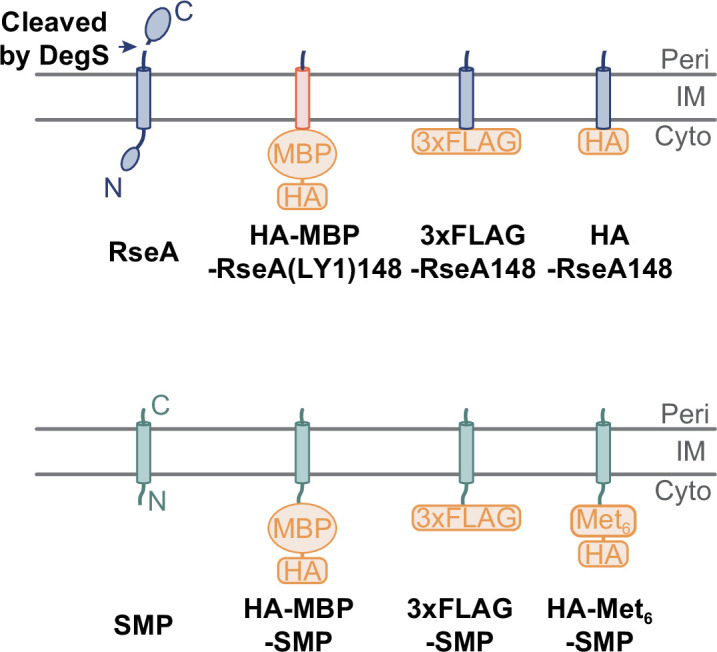
Schematic representation of the model substrates used in this study. Regions derived from RseA, SMP, and LY1 (the first TM of LacY) are shown in blue, green, and red, respectively. The HA-MBP, 3xFLAG, HA, and HA-Met_6_ tags are shown in orange. Peri, IM, and Cyto indicate periplasm, inner membrane, and cytoplasm, respectively.

To examine the possibility of RseP-dependent cleavage in *E. coli*, we arbitrarily selected 30 typical SMPs with approximately 50 or fewer amino acid residues. We also included seven proteins with larger sizes (68–161 amino acid residues). Although the latter proteins are larger than typical bacterial SMPs, we refer to them here as “SMP” for convenience, as they are also relatively small proteins. The TMHMM (32) or TOPCONS (33) program predicts that all these SMPs have a single membrane-spanning region. While the RseP/S2P substrates are generally single-spanning membrane proteins with type II (N_IN_ − C_OUT_) membrane topology (4, 7), some of the above 37 proteins were predicted to have type I (N_OUT_ − C_IN_) topology (Table S1). However, it has been suggested that certain small membrane proteins have a dual membrane topology (34). Furthermore, the computer-predicted topology does not always agree with the experimental results (34). Thus, as a first screening, we examined the *in vivo* cleavage of all the above 37 SMPs. We constructed derivatives with an N-terminal hemagglutinin (HA)-maltose-binding protein (MBP) tag (Fig. 1). The HA-MBP tag was expected to stabilize the cytoplasmic RseP cleavage products and enable their detection using antibodies against the HA tag. The HA-MBP tag has been successfully used to analyze other RseP substrates, such as RseA, FecR, signal peptides, and small membrane protein YqfG (12, 13, 20, 35).

**Fig 2:**
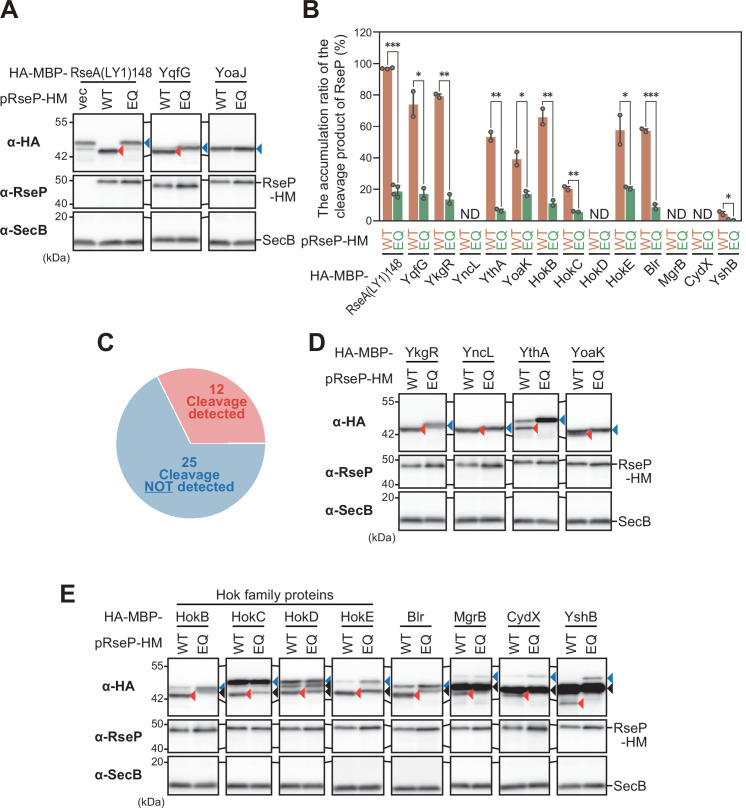
*In vivo* cleavage of N-terminal HA-MBP-tagged SMP by overproduced RseP. (**A–E**) RseP cleavability of HA-MBP-tagged model substrates. KA306 (Δ*rseA* Δ*rseP* Δ*clpP*) cells harboring pSTD689 (vector, vec), pYH9 [RseP-HM (His_6_-Myc), WT (wild-type)], or pYH13 [RseP(E23Q)-HM, EQ (E23Q)] were further transformed with a plasmid encoding an HA-MBP-RseA(LY1)148 (pYH20) or HA-MBP-SMP model substrate, as indicated. Deletion of the *clpP* gene, which encodes the protease subunit of the Clp proteases, stabilizes the RseP cleavage product of RseA. Cells were grown in L medium containing 1 mM isopropyl-β-D-thiogalactopyranoside (IPTG) at 30°C until the late log phase to induce both RseP-HM and HA-MBP-tagged model substrates. Acid-precipitated total proteins were analyzed by 7.5% Laemmli SDS-PAGE and anti-HA (α-HA) immunoblotting and by 12.5% Laemmli SDS-PAGE and anti-RseP (α-RseP), or anti-SecB (α-SecB) immunoblotting. SecB served as a loading control. Blue and red triangles indicate the full-length and the RseP-cleaved forms of the model proteins, respectively. A representative result from at least two biological replicates is shown. (A) RseP cleavability of control model substrates. (B) Cleavage efficiency of HA-MBP-tagged model substrates. The cleavage efficiencies were calculated as the ratio of the RseP-cleaved form to the total (full length plus cleaved) HA-MBP-tagged model substrates. Means of the data from at least two biologically independent experiments are plotted with SD and individual data. HA-MBP-SMP model substrates for which accurate quantification was not possible due to the very small difference in mobility between the full-length and RseP-cleaved bands were labeled as ND. A one-tailed Student *t*-test was used to compare the values between the groups. *P  <  0.05, **P  <  0.01, and ***P  <  0.001. (C) Summary of the screening of SMPs with the N-terminal HA-MBP tag for their cleavage by RseP *in vivo*. Out of 37 SMPs screened, 12 were found to be cleaved by RseP (see also Table 1 and Fig. S1 and S6). (D and E) RseP cleavage of the HA-MBP-SMP model substrates. The HA-MBP-SMPs shown in D generated a single RseP-dependent cleavage product, while those shown in E generated additional fragments (black triangles) that was produced RseP independently. The contrast of each immunoblotting image was adjusted to clearly show the cleavage product. A representative result from at least two biological replicates is shown. SD, standard deviation.

**Fig 3:**
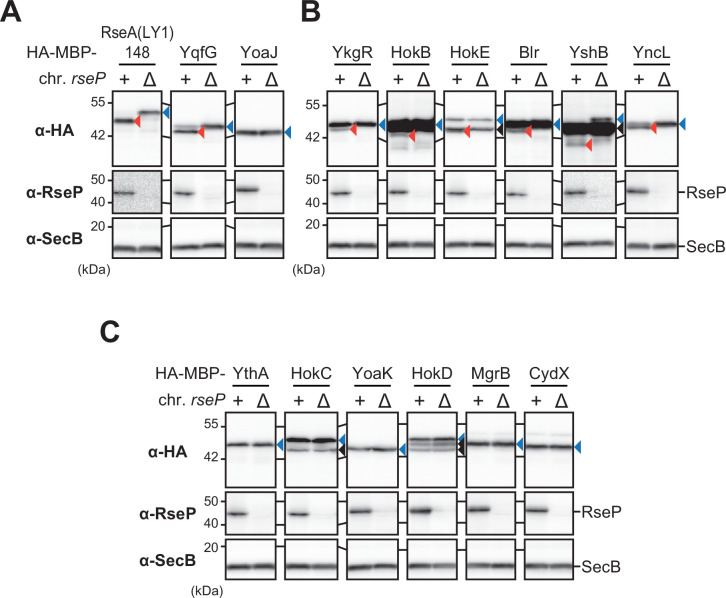
*In vivo* cleavage of SMPs with an N-terminal HA-MBP tag by chromosomally encoded RseP. (A) Cleavability of control model substrates by chromosomal-encoded RseP. KA304 (Δ*clpP* Δ*rseA rseP*^+^) or KA306 (Δ*clpP* Δ*rseA* Δ*rseP*) cells harboring pYH20 (HA-MBP-RseA(LY1)148, RseA(LY1)148), pEB82 (HA-MBP-YqfG, YqfG), or pEB74(HA-MBP-YoaJ, YoaJ) were grown and the proteins were analyzed, as shown in Fig. 2A. (B and C) Cleavage of the HA-MBP-SMP model substrates by chromosomally encoded RseP was examined as in A. The HA-MBP-SMPs shown in B generated an RseP-dependent cleavage product, whereas those shown in C did not. Blue, red, and black triangles indicate the full-length, RseP-cleaved, and RseP-independent-cleaved forms of the model proteins, respectively. The contrast of each immunoblotting image was adjusted to clearly show the cleavage products. A representative result from two biological replicates is shown.

We co-expressed the HA-MBP-tagged YqfG or RseA (LY1)148 [a derivative of a DegS-cleaved form of RseA with the first TM of LacY (LY1) in place of its original TM] with RseP from plasmids in a Δ*rseA* Δ*rseP* strain (although *rseP* is normally growth-essential, it can be deleted in the absence of *rseA*). As reported previously (15, 20, 35), they were efficiently cleaved to yield a stable N-terminal fragment that was detected with anti-HA antibodies when co-expressed with wild-type RseP (RseP-HM: C-terminal His_6_-Myc-tagged RseP), but not with its proteolytically inactive form [RseP(E23Q)-HM] [Fig. 2A and B; HA-MBP-RseA(LY1)148 and HA-MBP-YqfG]. We also confirmed that, in contrast to YqfG, little RseP-dependent cleavage was observed for an HA-MBP derivative of another SMP, YoaJ (Fig. 2A; HA-MBP-YoaJ), as previously reported (20). We then examined the possible RseP-dependent cleavage of HA-MBP derivatives of the 37 target SMPs. The results showed that 12 generated a smaller N-terminal fragment depending on the proteolytic activity of RseP (Fig. 2B and C; Fig. S1). For four of them (YkgR, YncL, YthA, and YoaK), a single cleavage product was detected in addition to the expected full-length proteins (Fig. 2B and D; Fig. S2). The other eight (HokB, HokC, HokD, HokE, Blr, MgrB, CydX, and YshB) also generated an RseP cleavage product. For these proteins, one or two additional bands of intermediate size (between those of the RseP cleavage product and the expected full-length protein) were generated independently of the RseP activity (Fig. 2B and E; Fig. S2), suggesting that they were cleaved by other proteases in addition to RseP. It is unclear whether the observed RseP cleavage occurred before or after cleavage by other proteases. Nevertheless, the results indicate that these 12 SMPs are potential novel proteolytic substrates of RseP. We then investigated whether chromosomally encoded RseP, rather than plasmid-expressed RseP, could cleave HA-MBP-SMP model substrates. The control model substrates, HA-MBP-RseA(LY1)148, HA-MBP-YqfG, and HA-MBP-YoaJ, were expressed in an *rseP*^+^ or Δ*rseP* strain and analyzed by anti-HA immunoblotting. For HA-MBP-RseA(LY1)148 and HA-MBP-YqfG, cleavage bands of apparently the same size as those observed in the RseP-overproduced condition were detected in a chromosomal *rseP* gene-dependent manner, but no such band was detected for HA-MBP-YoaJ (Fig. 3A), indicating that the chromosomally encoded RseP can cleave the former two proteins. Among the above 12 SMPs, six (YkgR, HokB, HokE, Blr, YshB, and YncL) also produced a degradation fragment only in the presence of the chromosomal *rseP^+^* gene (Fig. 3B), although no RseP-dependent fragment production was detected for the remaining six (Fig. 3C). These results strongly suggest that at least the former SMPs can be cleaved by chromosomally encoded RseP.

**Fig 4:**
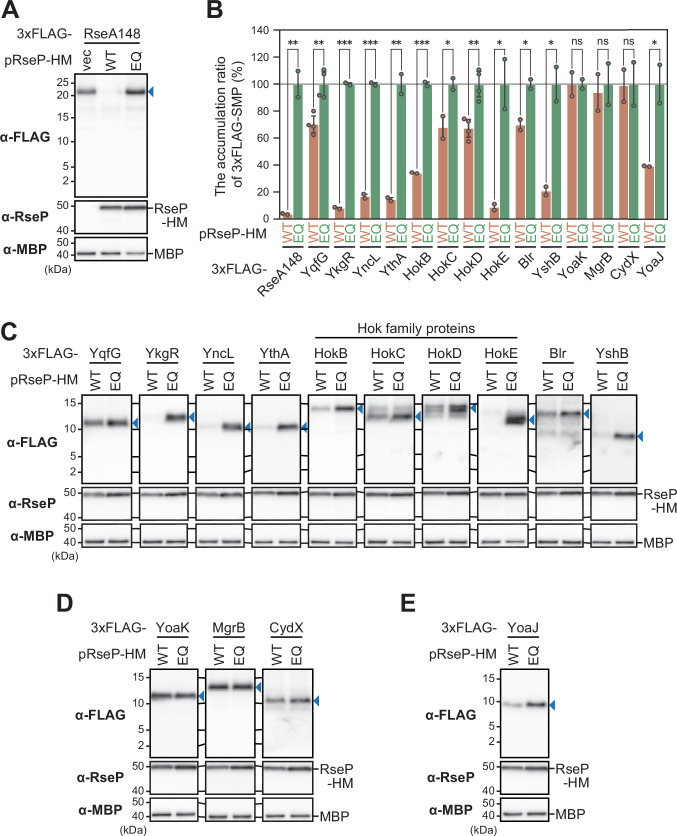
*In vivo* cleavage of SMPs with an N-terminal 3xFLAG-tag. RseP cleavability of 3xFLAG-RseA148 and 3xFLAG-SMP model substrates. KA306 (Δ*rseA* Δ*rseP* Δ*clpP*) cells harboring pSTD689 (vector, vec), pYH9 (RseP-HM, WT), or pYH13 (RseP(E23Q)-HM, EQ) were further transformed with a plasmid encoding 3xFLAG-RseA148 (pYK347) or 3xFLAG-SMP. RseP-dependent proteolysis was examined as shown in Fig. 2A except that the proteins were analyzed by 15% Bis-Tris SDS-PAGE and anti-FLAG (α-FLAG) or anti-MBP (α-MBP) immunoblotting. MBP served as a loading control. (A) RseP-dependent decrease in the accumulation level of 3xFLAG-RseA148. Blue triangle indicates the full-length form of 3xFLAG-RseA148. A representative result from two biological replicates is shown. (B) The ratio of the accumulation level of 3xFLAG-SMPs in cells expressing wild-type RseP-HM to that in cells expressing RseP(E23Q)-HM. Accumulation levels of the 3xFLAG-SMPs were normalized to the MBP signal, with the average accumulation level of 3xFLAG-SMPs in cells expressing RseP(E23Q)-HM set to 100%. Means of at least two biologically independent experiments are shown with SD and individual data. A one-tailed Student *t*-test was used to compare the values between the groups. *P  <  0.05, **P  <  0.01, ***P  <  0.001, and ns, not significant. (C) Ten 3xFLAG-SMP model substrates that exhibited significant RseP-dependent decrease in their accumulation level (*P* < 0.05) and (D) three 3xFLAG-SMP model substrates that exhibited almost no RseP-dependent decrease in their accumulation level. (E) RseP-dependent decrease in the accumulation level of 3xFLAG-YoaJ (*P* < 0.05). Blue triangles indicate the full-length forms of 3xFLAG-SMPs. A representative result from at least two biological replicates is shown in C–E. SD, standard deviation.

It is conceivable that the attachment of the relatively large HA-MBP tag (383 amino acid residues) affects the RseP susceptibility of SMPs *in vivo* by altering their membrane assembly, structure, and/or oligomerization state. Therefore, we next analyzed the cleavage of the SMPs with a smaller tag, 3xFLAG (22 amino acid residues). The 3xFLAG tag was used because of its small size and high specificity to the anti-FLAG antibody, which will help detect SMP derivatives. First, as a control, the model substrate RseA148 with an N-terminal 3xFLAG tag (3xFLAG-RseA148, 172 amino acids) (Fig. 1) was expressed with RseP in a Δ*rseP* background and analyzed by anti-FLAG immunoblotting with a 15% Bis-Tris gel, which allows the separation and detection of a small protein of larger than about 2 kDa as a sharp band (Fig. 4A). In this case, the N-terminal cleavage fragment of 3xFLAG-RseA148 was not detected, presumably due to its small size and/or instability (36, 37), and the accumulation level of the full-length form of this protein decreased drastically upon co-expression of wild-type RseP. In contrast, co-expression of the RseP(E23Q) mutant did not exert such an effect (Fig. 4A and B; Fig. S3). These results indicated that 3xFLAG-RseA148 was cleaved by RseP and confirmed that the N-terminal 3xFLAG tag could be used in the cleavage assay. We then constructed 3xFLAG-tagged derivatives of the 14 SMPs [12 identified as potential substrates in the first screening using the HA-MBP tag (Fig. 2), as well as YqfG and YoaJ] (3xFLAG-SMP, 49–76 amino acids) (Fig. 1). These SMP derivatives were co-expressed with RseP-HM or RseP(E23Q)-HM and analyzed using immunoblotting with anti-FLAG antibodies. Quantitation of the accumulation levels of the full-length form of each 3xFLAG-SMP showed that 10 of them (YqfG, YkgR, YncL, YthA, HokB, HokC, HokD, HokE, Blr, and YshB) exhibited a significant decrease depending on the RseP activity (Fig. 4B and C; Fig. S3), strongly suggesting that they are cleaved by RseP, although no significant decrease was observed for YoaK, MgrB, or CydX (Fig. 4B and D; Fig. S3). We also found that the accumulation level of 3xFLAG-YoaJ decreased in an RseP activity-dependent manner (Fig. 4B and E; Fig. S3), although we did not observe a clear cleavage of HA-MBP-YoaJ by RseP (Fig. 2A) (20). Although the results with HA-MBP-SMPs were not completely consistent with those with 3xFLAG-SMPs, the latter results support that several SMPs can become substrates of RseP in a near-native form.

### RseP directly cleaves SMPs *in vitro*

To validate the direct proteolysis of 14 potential SMP substrates (12 SMPs identified in the first screen plus YqfG and YoaJ) by RseP, we examined their *in vitro* cleavage using purified RseP. As it was difficult to purify these SMPs in sufficient quantities for an *in vitro* assay owing to their rather small sizes and high overall hydrophobicity, we synthesized them using the PURE system (38, 39), an established cell-free protein synthesis system. To improve the solubility of the SMPs and enable their sensitive detection, an HA-Met_6_ tag (19 amino acid residues) was attached to their N-terminus. The HA-Met_6_-SMPs were radiolabeled by synthesis in the presence of [^35^S]-methionine, and the translation mixtures containing a ^35^S-Met-labeled SMP were mixed with purified RseP and incubated at 37°C in the presence of *n*-dodecyl-*β*-D-maltoside, a non-ionic detergent, to analyze their cleavage by RseP. The wild type and the E23Q mutant forms of RseP proteins were purified by two-step chromatography: affinity purification using a C-terminal PA tag that is recognized with very high affinity by specific antibodies (40), and the following two rounds of size-exclusion chromatography after the proteolytic removal of the PA tag with tobacco etch virus (TEV) protease to obtain monodisperse RseP samples (Fig. S4).

**Fig 5:**
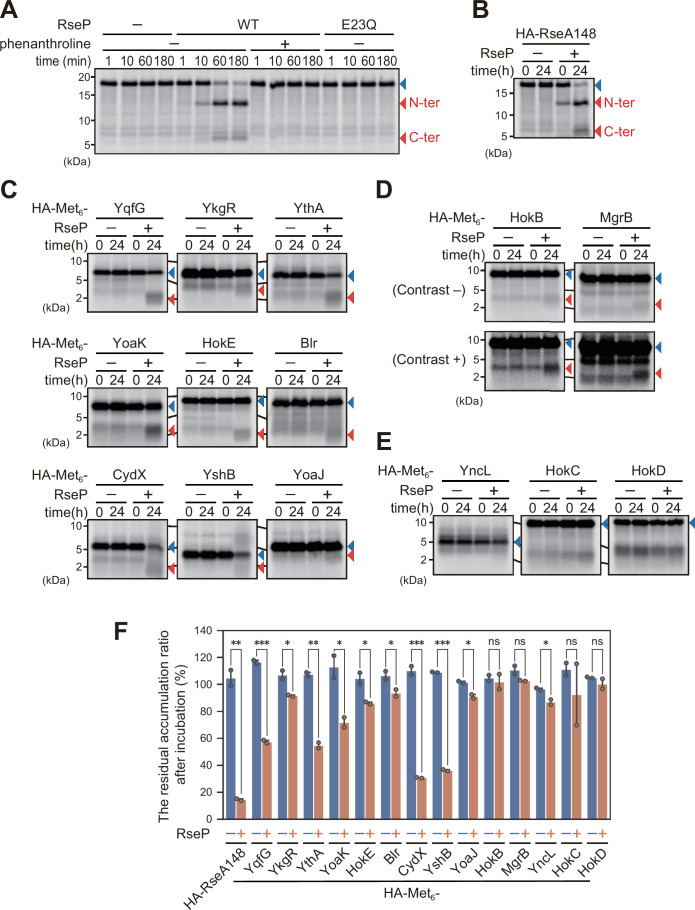
*In vitro* analysis of the HA-Met_6_-tagged SMP cleavage by RseP. (A) *In vitro* analysis of direct RseP-catalyzed cleavage of HA-tagged RseA148. The HA-RseA148 model substrate synthesized by the PURE system in the presence of ^35^S-Met was incubated with purified wild-type RseP (WT), RseP(E23Q)-HM (E23Q), or buffer only (−) in the presence or absence of 5 mM 1,10-phenanthroline (dissolved in dimethyl sulfoxide (DMSO); the final concentration of DMSO was 5%) for the indicated periods. The proteins in the reaction mixtures were analyzed by 15% Bis-Tris SDS-PAGE and phosphor imaging. The N-terminal (N-ter) or C-terminal (C-ter) cleaved fragment was predicted to contain 7 or 2 [^35^S]-labeled methionine, respectively. The full-length proteins and predicted RseP cleavage products are indicated by blue and red triangles, respectively. A representative result from two biological replicates is shown. (**B–E**) RseP-catalyzed cleavage of *in vitro* synthesized HA-RseA148 (B) and HA-Met_6_-SMPs (**C–E**) after 24 h incubation. HA-tagged RseA148 (B) and HA-Met_6_ tagged SMP proteins (**C–E**) were synthesized as in A and incubated at 37°C for the indicated periods with (+) or without (−) purified wild-type RseP. Total proteins in the reaction mixtures were analyzed as shown in A. SMPs for which cleavage products were detected are shown in C and D, and those for which cleavage products were not detected are shown in E. The lower panels labeled “Contrast+” in D are signal-enhanced images of the upper panels labeled “Contrast−”. A representative result from two biological replicates is shown in B–E. (F) The RseP-dependent decrease in signal of the full-length bands of SMPs. The percentage of the full-length signal after incubation (24 h) relative to that at the beginning of incubation (0 h) was calculated. Means of two independent experiments are shown with SD and raw data. A one-tailed Student *t*-test was used to compare the values between the groups. *P  <  0.05, **P  <  0.01, ***P  <  0.001, and ns, not significant. SD, standard deviation.

We first tested whether RseP-catalyzed substrate cleavage could be recapitulated in this system using *in vitro*-synthesized HA-RseA148 (note that a Met_6_ sequence had not been introduced into this substrate as HA-RseA148 contains nine Met residues in total) (Fig. 1, HA-RseA148). When the *in vitro* synthesized full-length HA-RseA148 was incubated with purified wild-type RseP, the amount of HA-RseA148 decreased over time with the concomitant generation of two bands whose apparent sizes match well with the sizes of the N- and C-terminal fragments of HA-RseA, that would be generated if RseP cleaved this protein at the expected site (between Ala-108 and Cys-109 of RseA) (35, 36) (Fig. 5A). No such fragments were generated in the presence of 1,10-phenanthroline, a zinc chelator that inhibits the activity of zinc metallopeptidases including RseP (17, 35), or when HA-RseA148 was incubated with the RseP(E23Q) mutant (Fig. 5A). These results strongly suggest that HA-RseA is cleaved by RseP. Together with our recent results obtained using a similar assay system (17), these results demonstrate that this *in vitro* assay system can be used to investigate substrate cleavage by RseP.

We then examined the RseP-catalyzed cleavage of the *in vitro*-synthesized HA-Met_6_-tagged forms of the 14 SMP substrates (Fig. 1, HA-Met_6_-SMP). To facilitate the detection of the RseP cleavage products for inefficiently cleaved proteins, we used a 10-fold amount of purified RseP as compared to that used in the experiment shown in Fig. 5A. We confirmed that with this amount of RseP, HA-RseA148 was efficiently cleaved by RseP (Fig. 5B). Among the 14 SMPs, nine (YqfG, YkgR, YthA, YoaK, HokE, Blr, CydX, YshB, and YoaJ) clearly gave a smaller band only in the presence of purified RseP (Fig. 5C). HokB and MgrB also produced a very small but significant amount of a smaller fragment in an RseP-dependent manner (Fig. 5D), while such a fragment was not reproducibly detected for the remaining three (YncL, HokC, and HokD) (Fig. 5E). We concluded that the 11 SMPs that gave an RseP-dependent fragment were cleaved by RseP. The quantification data showed that, among these 11 SMPs, the amount of the full-length proteins was significantly decreased upon incubation with RseP for those other than HokB and MgrB (Fig. 5F). Although the decrease in the amount of the full-length proteins was not evident for HokB and MgrB, we classified these two SMPs as being cleaved by RseP, as they produced RseP-dependent fragments. Taken together, these results demonstrate that RseP can directly cleave many of the SMPs examined.

### RseP can suppress the functions of HokB through its proteolytic elimination

We focused on the Hok proteins to investigate whether RseP-mediated cleavage of SMPs affects their biological functions. Among the 14 potential SMP substrates identified in this study, four were the Hok proteins of which two (HokB and HokE) were found to be cleaved by RseP in all four assay systems described above (three *in vivo* and one *in vitro*) (Fig. 2E, 3B, 4C, 5C and D; Table 1). Hok family proteins are intrinsic toxins of the type I toxin-antitoxin system (41). The *hok* genes were initially discovered as plasmid-borne genes involved in the maintenance of plasmids, such as plasmid R1 (42, 43), and subsequently, five *hok* family genes (*hokA-E*) were identified on the *E. coli* chromosomes (Fig. S5A) (27). Plasmid-encoded Hok proteins play a role in plasmid maintenance by killing plasmid-free segregants (42). In addition to the chromosomally encoded Hok proteins, we also found that an HA-MBP-tagged form of FlmA (a Hok-homolog encoded on an F plasmid and involved in its maintenance) (Fig. S5A) (44) was cleaved by RseP *in vivo* (Fig. S5B). The reason why cleavage of the HA-MBP-HokA was not detected among the chromosomally encoded Hok proteins in the first screening is unclear. However, it might be ascribed to the severe growth inhibition caused by its expression (Fig. S5C).

**TABLE 1:** Summary of the results for the RseP-catalyzed cleavages of SMPs^*a*^

	*In vivo*	*In vivo*	*In vivo*	*In vitro*	Class
N-terminus tag	HA-MBP	HA-MBP	3xFLAG	HA-Met_6_	
RseP	Plasmid borne	Chromosomally encoded	Plasmid borne	Purified enzyme	
RseA	+	+	+	+	
YqfG	+	+	+	+	Class I
YkgR	+	+	+	+	
HokB	+	+	+	+	
HokE	+	+	+	+	
Blr	+	+	+	+	
YshB	+	+	+	+	
YoaJ	−	−	+	+	Class II
YncL	+	+	+	−	
YthA	+	−	+	+	
YoaK	+	−	−	+	
HokC	+	−	+	−	
HokD	+	−	+	−	
MgrB	+	−	−	+	
CydX	+	−	−	+	

^*a*^ + and − indicate detection and non-detection of RseP-catalyzed cleavage, respectively.

Among the Hok proteins, the properties and functions of HokB have been studied in detail. After synthesis, HokB inserts into the cytoplasmic membrane and multimerizes to form small, membrane-embedded pores. These pores abrogate cell growth by causing the leakage of intracellular adenosine triphosphate (ATP) and other small molecules and by dissipating the proton motive force, leading to the inhibition of ATP synthesis (28 - 30). We examined the effects of RseP-mediated HokB cleavage on the HokB-induced cell phenotypes.

**Fig 6:**
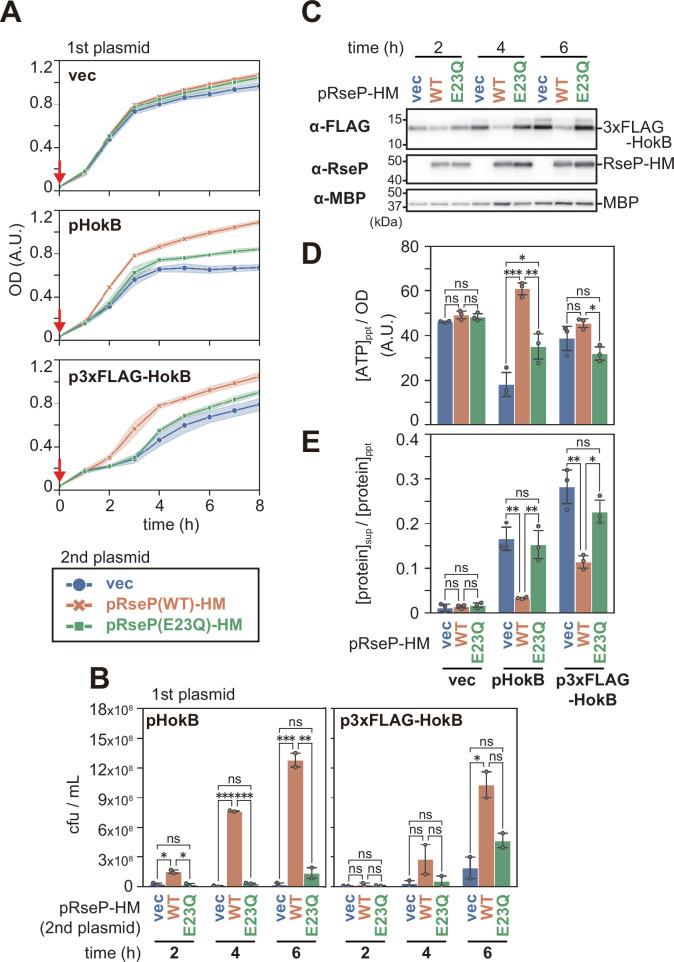
Suppression of the biological activities of HokB by RseP. (A and B) Suppression of HokB-induced growth defect by RseP. YK225 (Δ*hokB-175* Δ*rseA* Δ*rseP::kan*) cells harboring pTWV228 (vec, 1st plasmid), pYK99 (pHokB, 1st plasmid), or pYK78 (p3xFLAG-HokB, 1st plasmid) were further transformed with pSTD689 (vec, 2nd plasmid), pYH9 [pRseP(WT)-HM, 2nd plasmid], or pYH13 [pRseP(E23Q)-HM, 2nd plasmid]. *hokB, 3xflag-hokB,* and *rseP-hm* are placed under the control of the *lac* promoter on each plasmid. The cells were grown at 37°C in L medium supplemented with 1 mM IPTG and 1 mM cAMP to induce both HokB and RseP from the onset of cultivation (0 h, red arrows). (A) Optical density (OD) measured with Taitec mini photo 518R (660 nm) every 1 h. Means of data from at least four biologically independent experiments are shown with SD (light-colored shade). (B) The cfu measurement with HokB- and RseP-expressed cells. Cells were harvested at the 2-, 4-, and 6-h time points, diluted appropriately with saline, and plated on L solid medium containing glucose, which minimizes the expression of RseP and HokB from the *lac* promoter, and the number of colonies was counted. Means of data from two biologically independent experiments are shown with SD and individual data. One-way analysis of variance (ANOVA) with Tukey’s test was performed, *P  <  0.05, **P  <  0.01, ***P  <  0.001, and ns, not significant. (C) Cleavage of HokB by RseP. YK225 cells harboring pYK78 in addition to pSTD689 (vec), pYH9 (WT), or pYH13 (E23Q) were grown as in A (the growth curves are shown in Fig. S5D). At the 2-, 4-, and 6-h time points, 500 µL of the cultures was removed and subjected to SDS-PAGE and anti-FLAG, anti-RseP, and anti-MBP immunoblotting analysis as in Fig. 4A. A representative result from two biological replicates is shown. (D and E) Suppression of the decrease in the cellular ATP levels (D) and protein leakage/cell lysis (E) induced by HokB. The cells were grown for 4 h as in A, and then 500 µL of the cultures was centrifuged to obtain the cell and supernatant fractions. The supernatant fractions were filtered to remove the contaminated cells. (D) The relative concentrations of ATP in the cells were quantified using the luciferin-luciferase method. The relative ATP levels per cell [(ATP)_ppt_/OD] were calculated by dividing the relative ATP concentrations by the optical densities of the cultures at the sampling point. (E) The amount of the total proteins in each fraction was measured, and the ratio of the amount of total proteins in the supernatant [(protein)_sup_] to that in the precipitate [(protein)_ppt_] was plotted. Means of the data from three biologically independent experiments are shown with SD and individual data. One-way ANOVA with Tukey’s test was performed, *P  <  0.05, **P  <  0.01, ***P  <  0.001, and ns, not significant. SD, standard deviation.

First, we tested whether RseP could neutralize the cytotoxicity of HokB by monitoring the growth of HokB- and/or RseP-expressing cells (Fig. 6A) and by counting the colony forming units (cfu) (Fig. 6B) over time. It has been reported that HokB expression impairs the cell growth (27). Consistently, induction of HokB (without tag) alone from a plasmid in the Δ*rseP* Δ*hokB* strain caused a clear growth defect [Fig. 6A, compare vec (1st plasmid) and pHokB]. Co-expression of RseP-HM, but not RseP(E23Q)-HM, clearly restored cell growth (Fig. 6A and B, pHokB). Expression of RseP-HM or RseP(E23Q)-HM in the absence of HokB had little effect on cell growth [Fig. 6A, vec (1st plasmid)]. Similar results were obtained when 3xFLAG-HokB was used instead of untagged HokB (Fig. 6A and B, p3xFLAG-HokB). These results showed that proteolytically active RseP suppressed HokB cytotoxicity. Anti-FLAG immunoblotting analysis showed that the accumulation level of 3xFLAG-HokB in 3xFLAG-HokB-expressed cells was greatly reduced depending on the proteolytic activity of co-expressed RseP (Fig. 6C; Fig. S5D), suggesting that RseP cleaved HokB to neutralize its cytotoxicity. Expression of RseP(E23Q) also caused a small but significant growth recovery (Fig. 6A, pHokB and p3xFLAG-HokB), although it had little effect on the accumulation level of HokB (Fig. 6C, compare vec and E23Q). This is probably because the RseP(E23Q) mutant captures a fraction of the HokB molecules to prevent their oligomerization, thereby suppressing their deleterious effects, as this mutant retains the ability to interact with a substrate (20). We noticed that the growth of cells expressing 3xFLAG-HokB in the absence of RseP was transiently arrested from 1 to 3 h after HokB induction, but apparently resumed thereafter (Fig. 6A, p3xFLAG-HokB). We hypothesize that although 3xFLAG-HokB strongly inhibits the growth of most cells in an early growth phase, which was not clearly observed with the expression of untagged HokB with lower cytotoxicity (Fig. 6A, pHokB), a subpopulation of cells less affected by 3xFLAG-HokB expression continues to grow and dominates after 3 h of 3xFLAG-HokB induction. Although it remains unclear how these apparently “3xFLAG-HokB-tolerant” cells are generated and what their properties are, RseP-independent degradation of 3xFLAG-HokB by other proteases including DegQ might be induced in this growth resumption in a fraction of cells, as it has been suggested that DegQ cleaves HokB to induce the awakening of persister cells (29).

In the above experiments, it is not known whether RseP can cleave accumulated HokB, which would form oligomers or pores in the membrane, and abrogate its cytotoxicity, since RseP and HokB were induced simultaneously (Fig. 6A through C). To address this point, we constructed a system in which the expression of HokB and RseP can be controlled independently (Fig. S5E and F). We found that the strong induction of HokB alone from an *ara* promoter arrested the cell growth in an early growth phase as observed with the 3xFLAG-HokB expression in Fig. 6A, and that the cell growth resumed approximately 1 h after HokB expression was turned off (Fig. S5E*,* vec). This resumption of growth might also be attributed to a subpopulation of cells less affected by HokB expression. Also in this case, once the cells begin to grow, the cellular level of HokB would decrease due to dilution of the pre-accumulated HokB, which would promote further cell growth. Induction of RseP during growth arrest resulted in a faster recovery of cell growth depending on the proteolytic activity of RseP [Fig. S5E*,* pRseP(WT)-HM], raising the possibility that RseP can proteolytically eliminate HokB even after HokB has formed pores in the membrane. Neutralization of HokB toxicity is probably not a primary reason for the *rseP* essentiality since the deletion of the *hokB* gene in the *rseP*-deleted background does not improve the cell growth (Fig. S5G).

ATP leakage and membrane depolarization are caused by HokB pores, resulting in decreased intracellular ATP levels (28, 30). Next, we investigated the effect of RseP on the HokB-induced decrease in intracellular ATP levels. We harvested cells 4 h after induction of HokB and/or RseP and measured the intracellular ATP levels (i.e., the relative ATP concentration per cell) using the luciferin-luciferase assay method (Fig. 6D). As expected, the expression of HokB greatly decreased the intracellular ATP level. However, the ATP level was completely restored when RseP-HM, but not RseP(E23Q)-HM, was co-expressed (Fig. 6D, pHokB). The expression of wild-type RseP-HM or RseP(E23Q)-HM alone did not alter intracellular ATP levels [Fig. 6D, vec (1st plasmid)]. These results suggest that RseP prevents HokB-induced leakage and/or inhibition of ATP synthesis via HokB cleavage.

**Fig 7:**
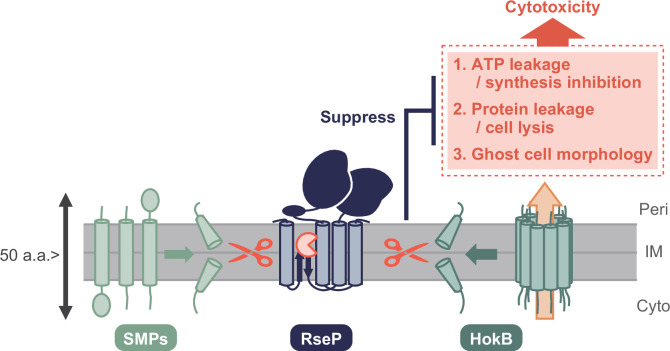
A model of RseP-catalyzed cleavage of SMPs and its functional significance. We have shown that RseP cleaves SMPs, including HokB, and also that RseP cleaves HokB to suppress the three phenotypes associated with HokB overexpression (i.e., ATP leakage and/or synthesis inhibition, protein leakage and/or cell lysis, and ghost cell morphology), which would contribute to the neutralization of the HokB cytotoxicity by RseP. Peri, IM, and Cyto indicate the periplasm, inner membrane, and cytoplasm, respectively.

Although similar results were obtained with 3xFLAG-HokB, the extent of recovery of intracellular ATP levels upon co-expression of wild-type RseP was much less (Fig. 6D, p3xFLAG-HokB). As mentioned above, it seems likely that a subpopulation of cells with lower sensitivity to the expression of 3xFLAG-HokB dominates at the 4-h time point. It is conceivable that such cells could significantly maintain the ATP levels, making the RseP dependence of the ATP levels unclear. These results suggest that RseP inhibits HokB pore formation via HokB cleavage, thereby preventing ATP leakage and/or inhibition of ATP synthesis (Fig. 7).

We speculated that the formation of HokB pores might not only decrease the cellular ATP levels, but also damage the inner membrane, resulting in protein leakage and/or cell lysis. To investigate this possibility, a portion of the culture was removed after 4 h of cell cultivation and fractionated into cell and supernatant fractions by centrifugation. The amount of total protein in each fraction was measured using the bicinchoninic acid (BCA) method. The degree of protein leakage and/or cell lysis was evaluated by dividing the amount of protein in the supernatant fraction by that in the cell fraction (Fig. 6E). We found that the expression of HokB or 3xFLAG-HokB induced protein leakage and/or cell lysis, which was suppressed by the co-expression of active RseP (Fig. 6E). These results suggest that cleavage of HokB by RseP suppresses HokB-induced protein leakage and/or cell lysis (Fig. 7).

Phase-contrast microscopy revealed the formation of cells with higher density pole regions and a lower density central region upon expression of HokB or 3xFLAG-HokB (Fig. S5H). This observation is consistent with a previous report that the cells expressing the Hok protein of R1 plasmid have a similar morphology, termed “ghost” (42). Co-expression of wild-type RseP significantly reduced the proportion of ghost cells (Fig. S5H), suggesting that RseP cleaves HokB to reduce cells with the ghost morphology. The density of the cytoplasm of ghost cells appeared to be lower than that of normal cells, which may be related to the protein leakage/cell lysis described above (Fig. 6E).

Taken together, we demonstrated that RseP cleavage of HokB suppresses the following three phenotypes associated with HokB overexpression: (i) leakage and/or synthesis inhibition of ATP, (ii) protein leakage and/or cell lysis, (iii) ghost cell formation (Fig. 7), which would contribute to the neutralization of the HokB cytotoxicity by RseP.

## DISCUSSION

Early studies of *E. coli* RseP demonstrated that this S2P family IMP regulates the σ^E^ extracytoplasmic stress response through cleavage of RseA and the maintenance of membrane quality through elimination of remnant signal peptides (3). Although it has been suggested that *E. coli* has additional substrates of RseP (12, 20), the apparent lack of consensus sequences/motifs in the known substrates makes it difficult to find candidates for the RseP substrate using a simple amino acid sequence search. A recent proteomic approach identified a novel substrate, FecR, that regulates the expression of the *fec* operon genes involved in ferric ion uptake (12). However, even this systematic approach has technical limitations in identifying hydrophobic membrane proteins, especially those with small molecular sizes, such as SMPs. This led us to focus on small membrane proteins based on their structural similarity to signal peptides known as RseP substrates. Our screening of SMPs resulted in the identification of several SMPs that could be cleaved by RseP.

Furthermore, the analysis of a possible SMP substrate, HokB, an endogenous toxin, showed that RseP suppresses HokB function by cleaving it (see below). These results suggest that SMPs are promising candidates for a new class of physiological substrates of RseP and raise the possibility that additional SMP substrate can be identified by more systematic analysis. Because the stability of SMPs and its impact on their functions are largely unknown, our results provide a novel perspective for studying SMPs. The discovery of additional RseP-cleavable proteins (14 SMPs) would also help to elucidate the requirements for RseP substrates. Further detailed analysis of the amino acid sequences of the substrates, including those identified in this study [e.g., by deep learning, together with the recently solved RseP structure and the proposed substrate-entry model (17)], could facilitate the identification of new substrates and our understanding of the molecular mechanism of RseP-catalyzed substrate cleavage.

We examined the RseP-dependent cleavage of 14 SMPs *in vivo* and *in vitro* using their derivatives with three different N-terminal tags: the HA-MBP tag (*in vivo*), the 3xFLAG tag (*in vivo*), and the HA-Met_6_ tag (*in vitro*). HA-MBP-tagged SMPs were cleaved not only by plasmid-expressed, but also by chromosomally encoded RseP *in vivo*. We found that of the 14 SMPs, six were cleaved in all of these assays (class I), although eight were cleaved under only two or three conditions (class II) (Table 1 and Fig. S6). The class II SMPs exhibited differential cleavability by RseP depending on the assay system, possibly because the tags fused to the SMPs altered their structure, particularly in their TMs and/or their oligomerization state, which would interfere with the recognition of the SMPs by RseP. In addition, tag fusion may affect the membrane orientation of SMPs. For example, a recent structural study revealed that CydX, a class II substrate candidate SMP, adopts a type I (N_OUT_ − C_IN_) topology in the solved structure of the cytochrome *bd*-I oxidase (45), although S2P peptidases, including RseP, are generally known to cleave type II (N_IN_ − C_OUT_) single-spanning membrane proteins (4, 7). This apparent discrepancy might result from the tag-induced reversion of the CydX topology, although it cannot be excluded that CydX can adopt dual topology depending on the situation, as has been suggested for several other SMPs (34). Although we understand that using tags would be potentially problematic, it is difficult to analyze the behavior of SMPs without the addition of tags, especially *in vivo*. Thus, it is important to examine the involvement of RseP activity in the functionality of candidate SMPs to establish that they are indeed a physiological substrate of RseP.

Among the candidate SMP substrates, HokB, whose function has been well characterized, was found to be cleaved under all the four assay conditions. Therefore, we investigated whether the proteolytic activity of RseP affects its function *in vivo*. Our results showed that RseP suppressed HokB-induced cytotoxicity, decrease in the intracellular ATP level, protein leakage/cell lysis, and ghost-cell morphology, depending on the proteolytic function of RseP (Fig. 6; Fig. S5). It has also been reported that HokB overexpression induces the formation of persisters, a small fraction of an isogenic population that is transiently tolerant to antibiotics (28 - 31). Thus, it is conceivable that RseP can negatively regulate HokB-induced persister cell formation through the cleavage of HokB. In addition, or alternatively, RseP may act positively in the awakening of persisters to resume growth by eliminating HokB, as our data suggest that RseP can abrogate the cytotoxicity of HokB even after its pore formation in the membrane (Fig. S5E and F). RseP would not directly cleave oligomerized HokB, as the intramembrane active site chamber of RseP is too narrow to accommodate an oligomerized HokB (17). RseP could cleave a monomeric HokB that has been dissociated from oligomers, resulting in a decrease in the number of cellular HokB molecules, and consequently in the number of HokB pores. It has been suggested that the periplasmic protease DegQ truncates the periplasmic domain of HokB to induce the awakening of persisters (29). We observed that HokB is cleaved by some protease *in vivo* (Fig. 2E)*,* probably in its periplasmic region, to generate a smaller fragment. By the analogy with the RseA-mediated extracytoplasmic stress response and the FecR-mediated regulation of ferric ion uptake, in which prior periplasmic cleavage(s) of these proteins triggers the subsequent RseP-catalyzed intramembrane proteolysis, it seems possible that RseP cleaves HokB after the processing of the HokB periplasmic region by DegQ and/or other proteases. The possible involvement of RseP in the regulation of persister formation/awakening is an interesting and important problem to be addressed, not only from a scientific but also from a medical point of view, as the persister formation is one of the most pressing problems in the treatment of bacterial infections caused by pathogenic bacteria (46). To investigate this possibility, sensitive and reliable experimental systems to investigate the formation and awakening of HokB-induced persisters should be established in future studies.

In addition to HokB, some candidate SMP substrates are reported to be involved in important cellular processes in *E. coli* and related enterobacteria. For example, Blr is thought to regulate cell division by stabilizing the divisome under certain stress conditions (47). It is also known that MgrB directly interacts with the sensor kinase PhoQ to modulate its activity, resulting in the deactivation of the PhoP/PhoQ two-component system (48). A homolog of YshB in pathogenic *Salmonella* promotes bacterial replication in host cells and regulates virulence (49). RseP-catalyzed cleavage of SMPs may also affect these cellular processes. It is possible that, in addition to the SMPs mentioned above, RseP is engaged in the functional regulation of other SMPs. It would be noteworthy that, in addition to Hok proteins, several other candidate SMP substrates such as Blr (class I), MgrB (class II), CydX (class II), and YshB (class I) were also found to be cleaved periplasmically by some unidentified proteases (Fig. 2E). The degradation of these SMPs may also be regulated by sequential cleavage by multiple proteases, including RseP. Further studies are needed to establish the RseP cleavage of the authentic form of these and other candidate SMP substrates and to understand its physiological significance.

This study demonstrated that RseP can cleave several SMPs, which would expand our knowledge about the substrates and possible cellular roles of RseP, and also shed light on a new aspect of the intracellular behavior of SMPs. In addition to RseP, several bacterial S2P peptidases, including *Bacillus subtilis* RasP (50 - 52), *Vibrio cholerae* YaeL (53, 54), and *Mycobacterium tuberculosis* Rip1 (55 - 57), have multiple kinds of substrates with no apparent amino acid sequence conservation, raising the possibility that some bacterial S2Ps have additional, still-unidentified substrates. It is reasonable to speculate that such substrates include SMPs, consistent with the previous finding that *B. subtilis* RasP can cleave a signal peptide (13) like *E. coli* RseP. S2Ps may also be involved in the degradation and regulation of SMPs in eukaryotic cells. These interesting and challenging possibilities deserve further investigation.

## MATERIALS AND METHODS

### The strains, plasmids, oligonucleotides, antibodies, and media

The *E. coli* K12 strains, plasmids, and oligonucleotides used in this study are listed in Table S2. Details of strain and plasmid construction, as well as the antibodies and media used are described in the Supplemental materials and methods.

### SDS-PAGE and immunoblotting

Total cellular proteins were precipitated with 5% trichloroacetic acid (TCA), washed with acetone, dissolved in SDS sample buffer, and analyzed by SDS-PAGE using a 7.5%, 10%, or 12.5% Laemmli gel or 15% Bis-Tris gel, and immunoblotting, essentially as described previously (12). The detailed procedure of immunoblotting is described in the Supplemental materials and methods.

### *In vivo* substrate cleavage assay

Details of the *in vivo* assays of the RseP-dependent cleavage of tagged-SMPs were carried out essentially as described previously (20). Briefly, cells were grown in L, induced with 1 mM IPTG for 3.5 h. Proteins were precipitated with 5% TCA and analyzed with SDS-PAGE and immunoblotting with appropriate antibodies.

### *In vitro* cleavage of SMPs by purified RseP

Details of production and purification of the wild type and the E23Q mutant form of RseP, as well as the *in vitro* cleavage assay procedures, are described in the Supplemental materials and methods.

### Growth assay of cells expressing HokB and RseP

For the HokB/RseP simultaneous expression system, *hokB*, *3xflag-hokB*, and *rseP-hm* were cloned under the *lac* promoter on compatible plasmids. Cells carrying appropriate combinations of plasmids were grown overnight in L medium containing 0.4% glucose (glucose was included to minimize a leaky expression of the proteins). The cells were then diluted 100-fold and grown at 37°C in L medium supplemented with 1 mM IPTG and 1 mM cAMP to induce HokB and RseP simultaneously. cAMP was added to the medium to maximize expression from the *lac* promoter as cAMP binds to catabolite activator protein, an activator of the *lac* promoter. Optical density (OD) was measured every 1 h using mini photo 518R (660 nm)(Taitec, Saitama, Japan). At the 2-, 4-, and 6-h time points, 200 µL of the culture was sampled, diluted appropriately with saline, and plated onto L agar plates containing 0.4% glucose. Plates were incubated overnight at 30°C, and the number of the colonies was counted to calculate the cfu.

For the HokB/RseP-independent expression system (Fig. S5E and F), *hokB* and *rseP-hm* were cloned under the *araBAD* and *lac* promoters, respectively, of compatible plasmids. Cells carrying appropriate combinations of plasmids were grown overnight in L-glucose medium as described above. They were then diluted 100-fold and grown at 37°C in L medium supplemented with 0.2% L-arabinose to induce HokB only. Two hours after the start of cultivation, the cells were washed three times with L medium supplemented with 0.05% D-fucose, a non-metabolizable L-arabinose analog that competitively inhibits expression from the *araBAD* promoter (58), resuspended in L medium supplemented with 1 mM IPTG and 0.05% D-fucose to induce only RseP-HM, and grown at 37°C. OD was measured every 1 h using mini photo 518R (660 nm)(Taitec , Saitama, Japan).

### Protein leakage/cell lysis analysis

Cells were grown as described for the growth assay for the simultaneous expression system. Five hundred microliters of the cultures was withdrawn at the 4-h time point, kept on ice for 5 min, and centrifuged at 4°C for 10 min at 10,000 rpm to obtain the cells and supernatant. The cells were resuspended in the same volume of 10 mM Tris-HCl (pH 8.1). The supernatants were filtered through a 0.22 µm polyvinylidene difluoride (PVDF) centrifugal filter (Merck, Darmstadt, Germany). Proteins in each fraction were precipitated with 5% TCA, washed with acetone, and solubilized in buffer containing 50 mM Tris-HCl (pH 8.1) and 5% SDS. One hundred microliters of the samples was mixed with 100 µL of Pierce BCA assay kit working reagent (Thermo Fisher Scientific, Waltham, MA, USA) in a transparent 96 well plate. After incubation at 37°C for 30 min and then at room temperature for 5 min, the absorbance at 562 nm was measured using the Viento Nano microplate reader (BioTek Instruments, Winooski, VT, USA). Albumin was used to obtain a calibration curve to determine the absolute amount of the proteins.

### ATP assay

The cell fraction was prepared as described for the protein leakage/cell lysis analysis. One hundred microliters BacTiter-Glo reagent (Promega, Madison, WI, USA) was mixed with 100 µL of the fraction in a black 96 well plate, incubated at 25°C and subjected to bioluminescence detection using Luminescent image analyzer LAS3000mini (Cytiva, Marlborough, MA, USA).

### Microscopic observations

Cells were grown as described for the growth assay of cells expressing HokB and RseP (simultaneous expression system), harvested at the indicated time points, spotted onto an M9 agar-pad containing 0.9% agarose on a glass slide, and observed under a bright-field phase-contrast microscope (BX53 equipped with 100X/1.30 NA objective, Olympus , Tokyo, Japan). Cell images were captured with Zyla 5.5 sCMOS camera (Andor, Belfast, Northern Ireland) and processed using MetaVue (Molecular Devices, San Jose, CA, USA) and Photoshop (Adobe, San Jose, CA, USA) software.

## SUPPLEMENTAL MATERIAL

Fig. S1 mbio.01086-23-s0001.pdf Screening of the SMPs cleaved by RseP.

Fig. S2 mbio.01086-23-s0002.pdf The original data of the RseP catalyzed cleavage of N-terminal HA-MBP-tagged SMP, shown in Fig. 2A, D, and E.

Fig. S3 mbio.01086-23-s0003.pdf Accumulation levels of RseP-HM coexpressed with a 3xFLAG-SMP.

Fig. S4 mbio.01086-23-s0004.pdf Purification of wild-type RseP and the E23Q mutant.

Fig. S5 mbio.01086-23-s0005.pdf Analysis of Hok family proteins.

Fig. S6 mbio.01086-23-s0006.pdf The amino acid sequences of RseP-cleavable SMPs.

Supplementary data mbio.01086-23-s0007.docx Supplemental materials and methods

Table S1 mbio.01086-23-s0008.xlsx Screened SMPs.

Table S2 mbio.01086-23-s0009.docx Strains, plasmids, and oligonucleotides.

ASM does not own the copyrights to Supplemental Material that may be linked to, or accessed through, an article. The authors have granted ASM a non-exclusive, world-wide license to publish the Supplemental Material files. Please contact the corresponding author directly for reuse.
