# Supplementary material for: S2P intramembrane protease RseP degrades small membrane proteins and suppresses the cytotoxicity of intrinsic toxin HokB
Source: mBio. 2023 Jul 6;14(4):e01086-23. doi: 10.1128/mbio.01086-23 (PMC10470546; doi:10.1128/mbio.01086-23)
Supplement: Fig. S1 — Screening of the SMPs cleaved by RseP. [file mbio.01086-23-s0001.pdf]

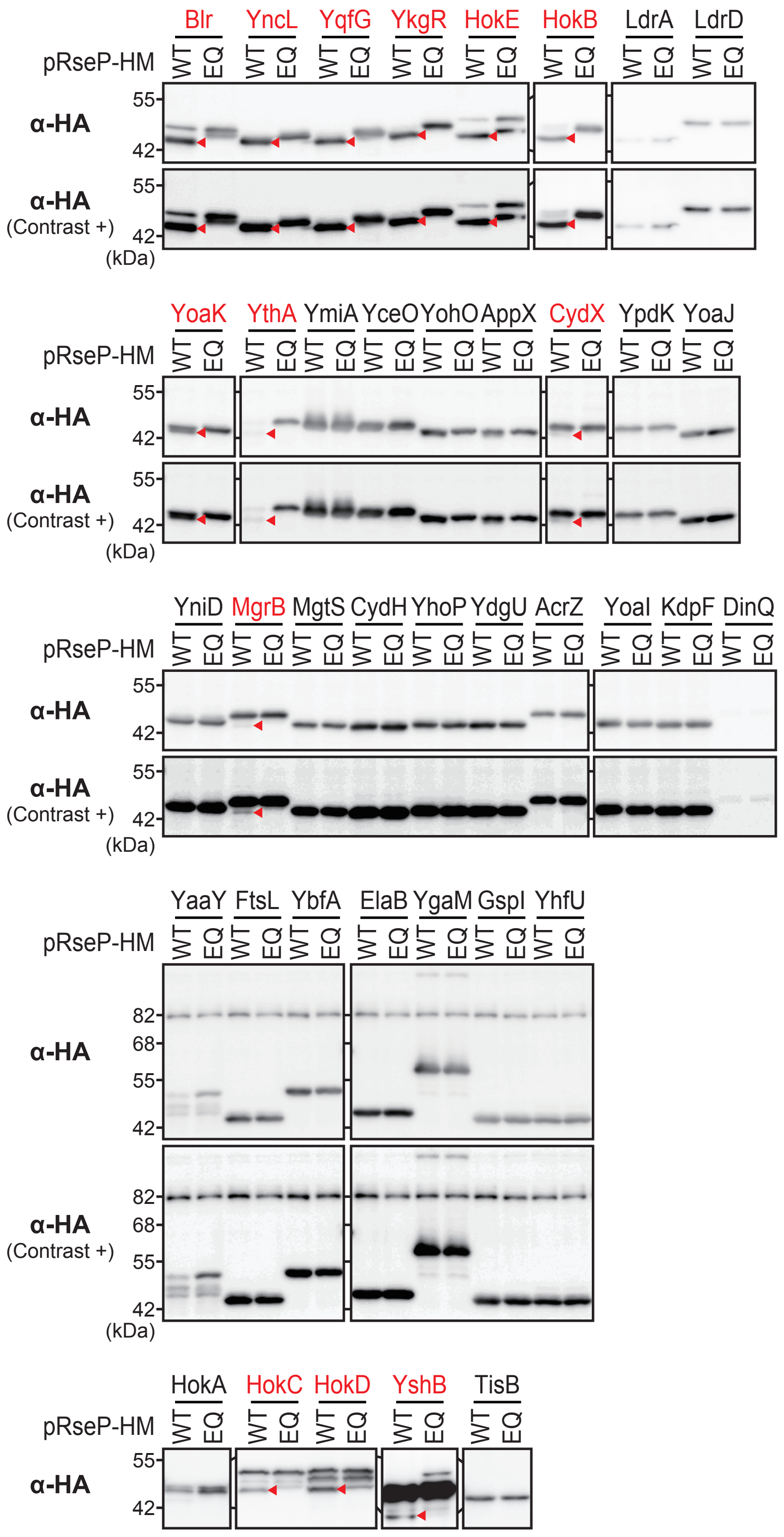

FIG S1 Screening of the SMPs cleaved by RseP. KA306 (Δ*rseA* Δ*rseP* Δ*clpP*) cells harboring pYH9 (wild-type RseP-HM, WT) or pYH13 [RseP(E23Q)-HM, EQ] were further transformed with a plasmid encoding an N-terminal HA-MBP-tagged SMP. Cells were grown and analyzed, as shown in Fig. 2A. Red triangles indicate the RseP cleavage products. The names of the SMPs that generated the RseP cleavage products are shown in red. The lower panels indicated by "Contrast +" are signal-enhanced images of the upper panels. A representative result from two biological replicates is shown.
