# Supplementary material for: S2P intramembrane protease RseP degrades small membrane proteins and suppresses the cytotoxicity of intrinsic toxin HokB
Source: mBio. 2023 Jul 6;14(4):e01086-23. doi: 10.1128/mbio.01086-23 (PMC10470546; doi:10.1128/mbio.01086-23)
Supplement: Fig. S2 — The original data of the RseP catalyzed cleavage of N-terminal HA-MBP-tagged SMP, shown in Fig. 2A, D, and E. [file mbio.01086-23-s0002.pdf]

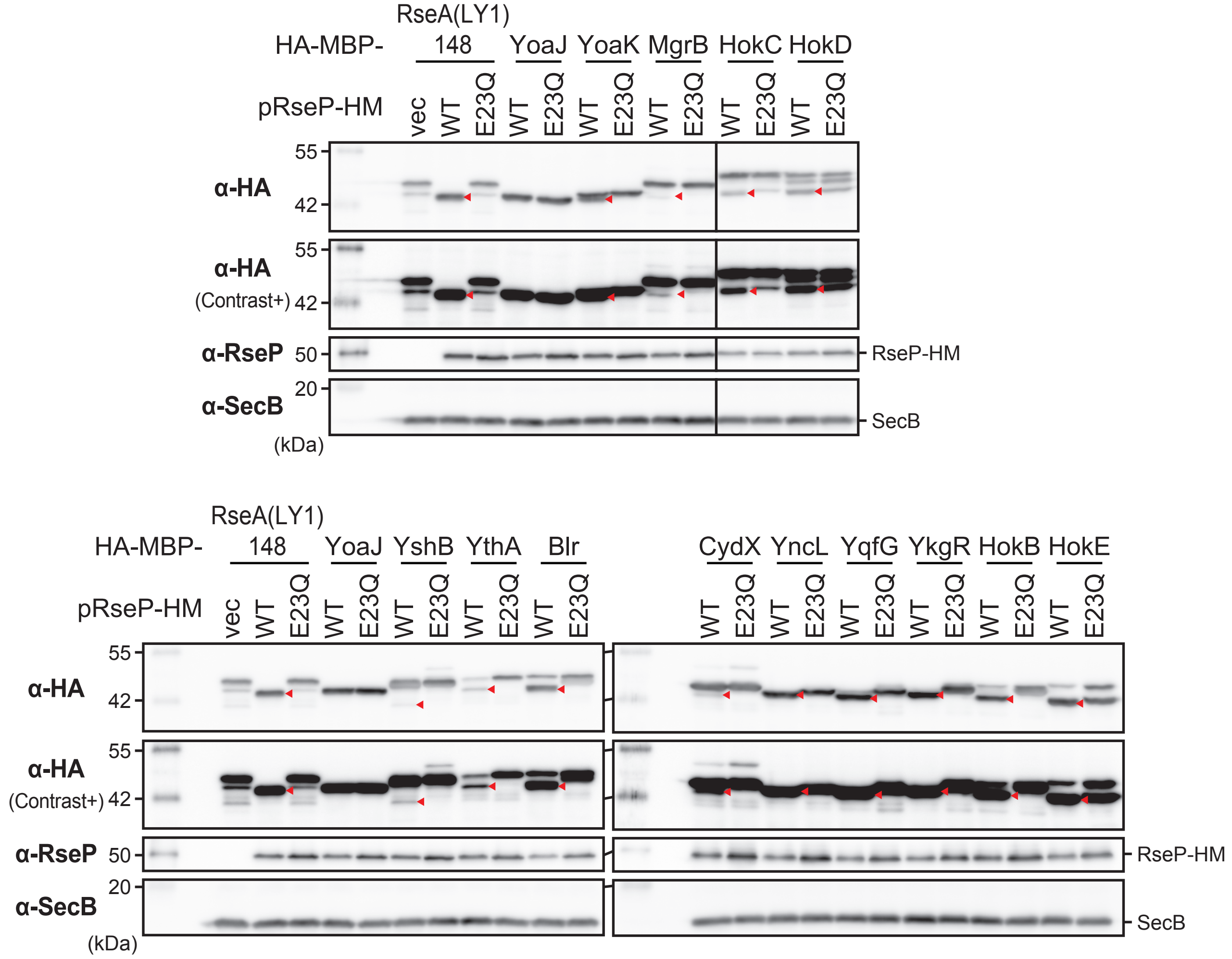

FIG S2 The original data of the RseP catalyzed cleavage of N-terminal HA-MBP-tagged SMP, shown in Fig. 2A, D, and E. KA306 (Δ*rseA* Δ*rseP* Δ*clpP*) cells harboring pSTD689 (vector, vec), pYH9 (RseP-HM, WT), or pYH13 [RseP(E23Q)-HM, E23Q] were further transformed with a plasmid encoding an HA-MBP-RseA(LY1)148 (pYH20) or HA-MBP-SMP model substrate. Cells were grown, analyzed ,and shown, as in Fig. S1. A representative result from two biological replicates is shown. The data shown in Fig. S2 represents the original figures of the data used in Fig. 2A, D, and E. In Fig. 2A, D, and E, the contrast was adjusted to clearly visualize the cleavage bands.
