# Supplementary material for: S2P intramembrane protease RseP degrades small membrane proteins and suppresses the cytotoxicity of intrinsic toxin HokB
Source: mBio. 2023 Jul 6;14(4):e01086-23. doi: 10.1128/mbio.01086-23 (PMC10470546; doi:10.1128/mbio.01086-23)
Supplement: Fig. S3 — Accumulation levels of RseP-HM coexpressed with a 3xFLAG-SMP. [file mbio.01086-23-s0003.pdf]

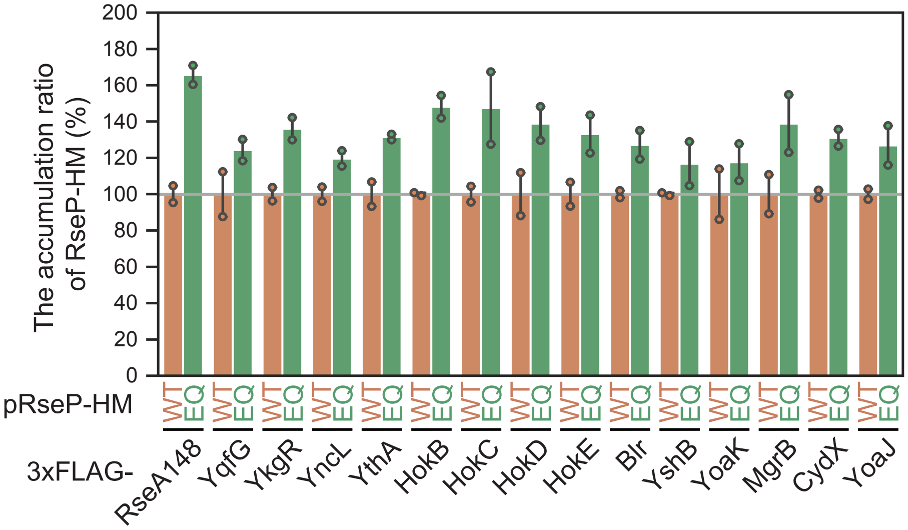

FIG S3 Accumulation levels of RseP-HM coexpressed with a 3xFLAG-SMP. Comparison of the accumulation levels of wild-type RseP-HM and the RseP(E23Q)-HM mutant coexpressed with the 3xFLAG-tagged model substrates. KA306 (Δ*rseA* Δ*rseP* Δ*clpP*) cells harboring pYH9 (RseP-HM, WT) or pYH13 [RseP(E23Q)-HM, EQ] were further transformed with a plasmid encoding 3xFLAG-RseA148 (pYK347) or 3xFLAG-SMP. Cells were grown and analyzed as shown in Fig. 4A. Accumulation levels of the RseP-HM and RseP(E23Q)-HM were normalized to the MBP signals, and then normalized to the average accumulation level of wild-type RseP-HM, which was set to 100%. Means of two biologically independent experiments are shown with SD and individual data. RseP(E23Q)-HM accumulated at an increased level as compared to wild-type RseP. Although the exact reason for this is unclear, it appears to be due to increased stability of RseP(E23Q)-HM in the cells.
