# Supplementary material for: S2P intramembrane protease RseP degrades small membrane proteins and suppresses the cytotoxicity of intrinsic toxin HokB
Source: mBio. 2023 Jul 6;14(4):e01086-23. doi: 10.1128/mbio.01086-23 (PMC10470546; doi:10.1128/mbio.01086-23)
Supplement: Fig. S4 — Purification of wild-type RseP and the E23Q mutant. [file mbio.01086-23-s0004.pdf]

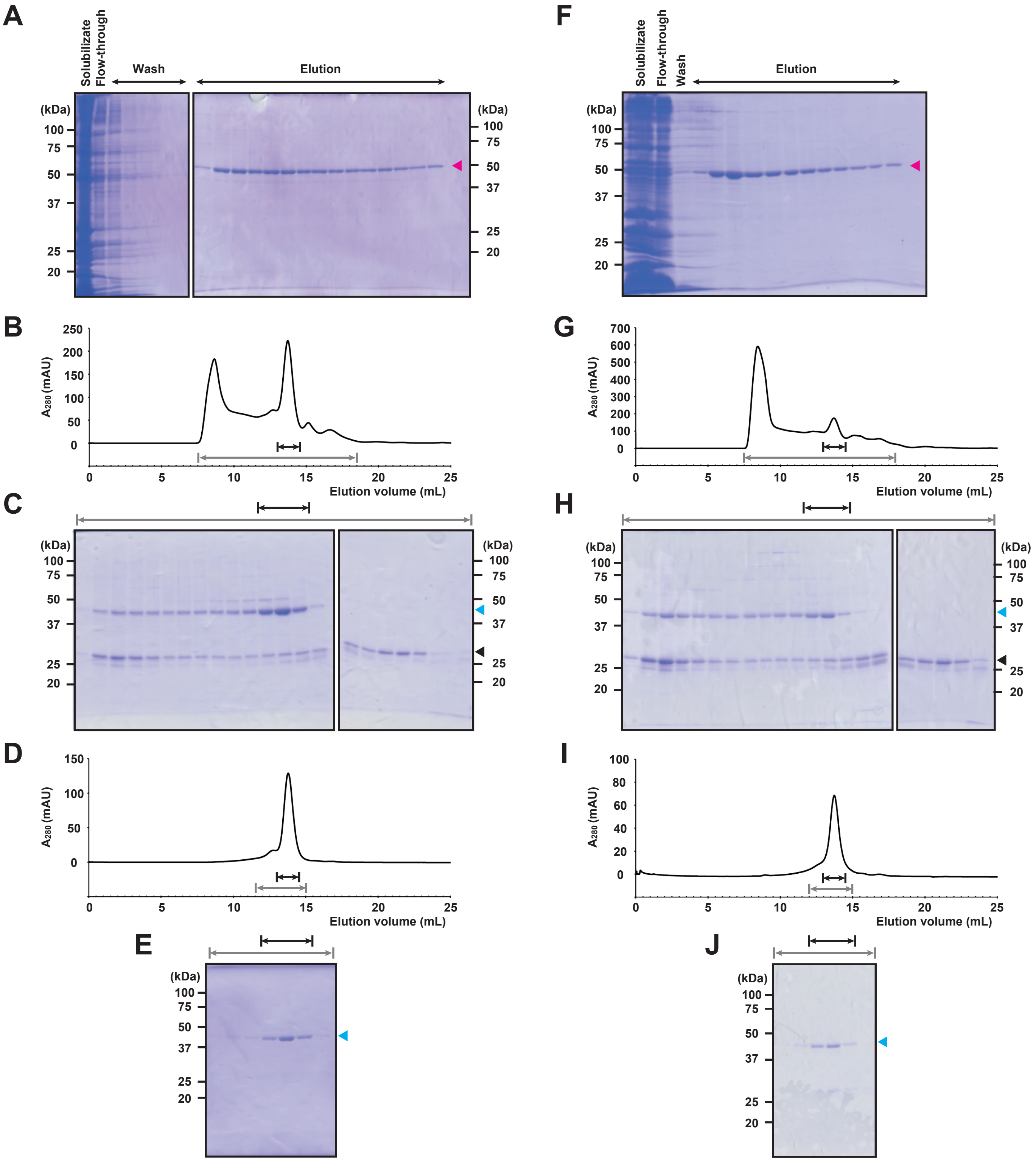

FIG S4 Purification of wild-type RseP and the E23Q mutant. (A-E) SDS-PAGE images and chromatograms for RseP(WT). (A) SDS-PAGE of the fractions from immunoaffinity purification using NZ-1 Sepharose. "Solubilizate" represents the supernatant after ultracentrifugation of the membrane fraction solubilized with sucrose monododecanoate. “Flow-through” represents the unbound fraction of the NZ-1 Sepharose affinity chromatography. The bands of RseP with a C-terminal tag are indicated by a magenta triangle. (B) The chromatogram of the first round of SEC. The fractions indicated with a gray double arrow were subjected to SDS-PAGE in (C). The fractions indicated with a black double arrow were collected and subjected to the second round of SEC in (D, E). Due to the tag cleavage by TEV protease, RseP appeared as lower molecular weight bands (cyan triangle) than those in (A). The bands of TEV protease are marked with a black triangle. (C) The SDS-PAGE images of the fractions from the first round of SEC. The lanes indicated with gray and black arrows correspond to the fractions shown in (B). The chromatogram (D) and the SDS-PAGE image (E) of the second round of SEC are shown as in (B) and (C), respectively. The bands of RseP are indicated with a cyan triangle. The fractions indicated by the black double arrow were collected and used for the in vitro cleavage assay. (F-J) SDS-PAGE images and chromatograms for RseP(E23Q) are shown as for WT in (A-E).
