# Supplementary material for: S2P intramembrane protease RseP degrades small membrane proteins and suppresses the cytotoxicity of intrinsic toxin HokB
Source: mBio. 2023 Jul 6;14(4):e01086-23. doi: 10.1128/mbio.01086-23 (PMC10470546; doi:10.1128/mbio.01086-23)
Supplement: Fig. S5 — Analysis of Hok family proteins. [file mbio.01086-23-s0005.pdf]

A

Uniprot ID	Protein name		Sequence	
P11895	Hok(plasmid R1)	1	MKLPRSSLVWCVLIVCLTLLIFTYLTRKSLCEIRYRDGHREVAAFMAYESGK	52
P62670	FlmA(F plasmid)	1	MKLPRSSLVWCVLIVCLTLLIFTYLTRKSLCEIRYRDGYREVAAFMAYESGK	52
P37305	HokA	1	MPQ--KYRLLSLIVICFTLLFFTWMIRDSLCELHIKQESYELAAFLACKLKE	50
P77494	HokB	1	MKH--NPLVVCLLIICITILTFTLLTRQTLYELRFRDGDKEVAALMACTSR-	49
P0ACG4	HokC	1	MKQ-HKAMIVALIVICITAVVAALVTRKDLCEVHIRTGQTEVAVFTAYESE-	50
P0ACG6	HokD	1	MKQ-QKAMLIALIVICLTVIVTALVTRKDLCEVRIRTGQTEVAVFTAYEPEE	51
P77091	HokE	1	MLT--KYALAAVIVLCLTVLGFTLLVGDSLCEFTVKERNIEFKAVLAYEPKK	50

■ 100% match
■ ≥50% match

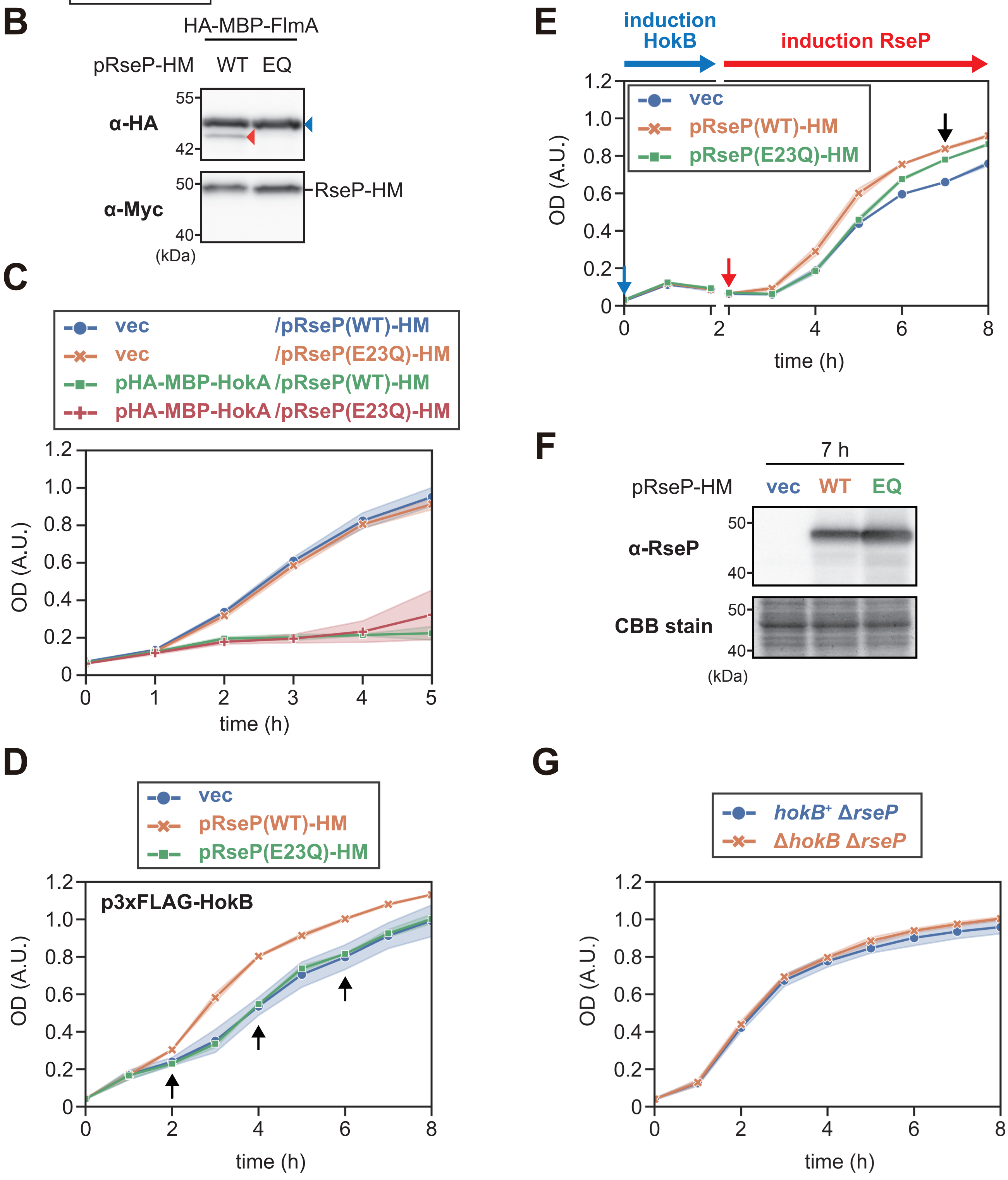

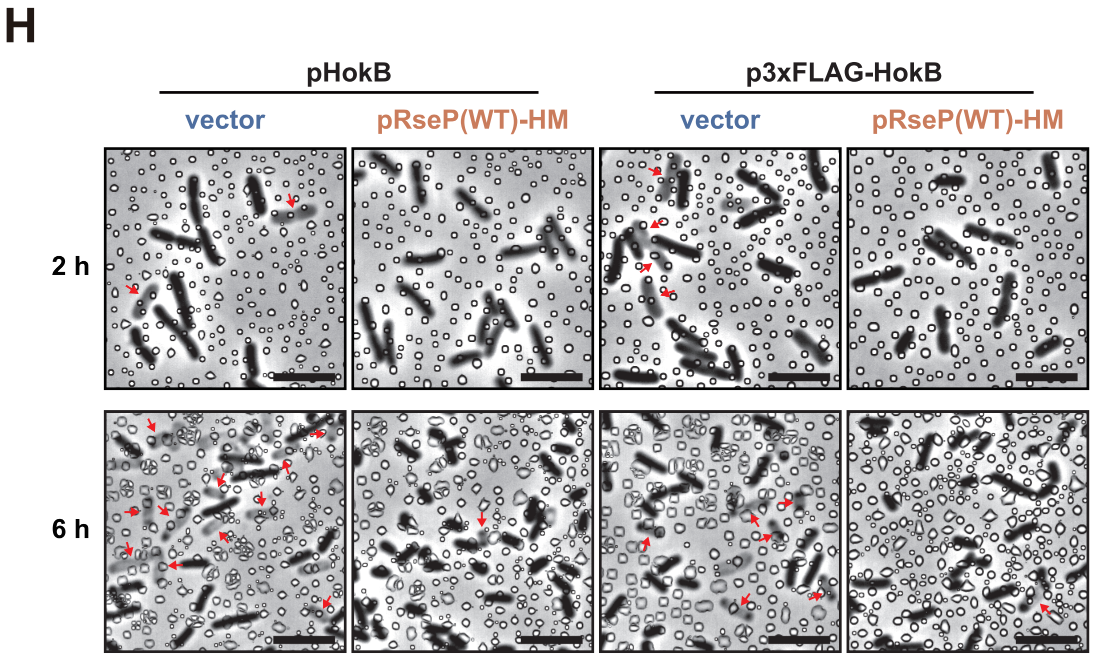

FIG S5 Analysis of Hok family proteins. (A) The amino acid sequence similarity of the Hok family proteins analyzed in this research. Amino acid sequences of *E. coli* Hok proteins were aligned using the Clustal W ver. 2.1 program (http://www.clustal.org/clustal2). Uniprot IDs, protein names, and sequences are shown. Conserved (100% match) and similar (≥ 50% match) residues are boxed in black and gray, respectively. (B) *In vivo* cleavage of HA-MBP-FlmA by RseP. KA306 (Δ*rseA* Δ *rseP* Δ*clpP*) cells harboring pYH9 (RseP-HM, WT) or pYH13 [RseP(E23Q)-HM, EQ] were transformed with pYK4 (HA-MBP-FlmA). Cells were grown, and proteins were analyzed by anti-HA or anti-Myc immunoblotting as shown in Fig. 2A. Blue and red triangles indicate the full-length proteins and N-terminal cleavage products, respectively. A representative result from two biological replicates is shown. (C) The severe growth inhibition caused by the expression of HA-MBP-HokA. KA306 cells harboring pYH9 or pYH13 were transformed with pTWV228 (vec) or pEB98 (HA-MBP-HokA). Cells were grown as in Fig. 2A, and the optical density (OD) was measured as shown in Fig. 6A. Means from three biologically independent experiments are shown with SD (light-colored shade). (D) Suppression of the HokB-induced cytotoxicity by RseP. YK225 (Δ*hokB-175* Δ*rseA* Δ*rseP*) cells harboring pYK78 (3xFLAG-HokB) were further transformed with pSTD689 (vec), pYH9 [RseP-HM, pRseP(WT)-HM], or pYH13 [RseP(E23Q)-HM, pRseP(E23Q)-HM]. Cells were grown, and the OD was measured as shown in Fig. 6A. Five hundred microliters of the culture were collected at 2, 4, and 6 h time points (red arrows), and proteins were analyzed using anti-FLAG, anti-RseP, and anti-MBP immunoblotting (Fig. 6C). A representative result from two biological replicates is shown with SD (light-colored shade). (E and F) RseP can potentially cleave HokB after its oligomerization to form pores and suppress its function. (E) YK225 (Δ*hokB-175* Δ*rseA* Δ*rseP*::*kan*) cells harboring pYK412, which encodes untagged HokB under the control of the *araBAD* promoter, were further transformed with pSTD689 (vector, vec), pYH9 [RseP-HM, pRseP(WT)-HM], or pYH13 [RseP(E23Q)-HM, pRseP(E23Q)-HM], in which *rseP-hm* is placed under the *lac* promoter. Cells were grown at 37 °C in L medium supplemented with 0.2 % arabinose to induce only HokB from the start of cultivation (0 h, blue arrow). The turbidity of the cultures plateaued after 1 h, indicating that the growth of these cells was inhibited by the HokB expression. At the 2 h time point, the cells were washed with L medium containing 0.05% fucose, which inhibits the expression of the *araBAD* promoter, and resuspended in L medium containing 1 mM IPTG and 0.05% fucose to induce only RseP-HM and resume the cell growth (2 h, red arrow). OD was measured every 1 h using Taitec mini photo 518R (660 nm) and means of two biologically independent experiments are shown with SD (light-colored shade). (F) Three hundred microliters of culture was collected at the 7 h time point (black arrow), and proteins were analyzed by anti-RseP immunoblotting and CBB staining to determine the accumulation levels of RseP-HM and RseP(E23Q)-HM. (G) The effect of chromosome-encoded HokB on the growth of Δ*rseP* cells. YK191 (*hokB*$^+$ Δ*rseA* Δ*rseP*::*kan*) and YK225 (Δ*hokB-175* Δ*rseA* Δ*rseP*::*kan*) cells were grown at 37 °C in L medium and OD was measured every 1 h. Means from three biologically independent experiments are shown with SD (light-colored shade). (H) Observation of cell morphology by phase-contrast microscopy. YK225 (Δ*hokB-175* Δ*rseA* Δ*rseP*::*kan*) cells harboring pYK99 (pHokB), or pYK78 (p3xFLAG-HokB) were further transformed with pSTD689 (vec), or pYH9 [pRseP(WT)-HM] and grown as shown in Fig. 6A. Cells were harvested at the 2 h and 6 h time points and their images on a 0.9% agar-pad were obtained using a bright-field phase-contrast microscope. Red arrow indicates “ghost cells”. Representative examples are shown. Scale bar, 5 μm. The small white circles in the photos are condenstaion on the camera lens.
