# Supplementary material for: S2P intramembrane protease RseP degrades small membrane proteins and suppresses the cytotoxicity of intrinsic toxin HokB
Source: mBio. 2023 Jul 6;14(4):e01086-23. doi: 10.1128/mbio.01086-23 (PMC10470546; doi:10.1128/mbio.01086-23)
Supplement: Fig. S6 — The amino acid sequences of RseP-cleavable SMPs. [file mbio.01086-23-s0006.pdf]

	Name	size (a.a.)	N-terminal	predicted TM	C-terminal
	RseA148	148	~WQKVRP	WAAQLTQMGVAACVSLAVIVG	VQHYNGQSETSQQPETPVFNTLPMMGKASPV
Class I	YqfG	41	MNFLMR	AIFSLLLLFTLSIPVISDCVAMA	IESRFKYMMLLF
Class I	YkgR	33	MKENKVQQISHK	LINIVVFVAIVEYAYLFLHF	Y
Class I	HokB	49	MKHNPL	VVCLLIICITILTFTLLTRQTLY	ELRFRDGDKEVAALMACTSR
Class I	HokE	50	MLTK	YALAAVIVLCLTVLGFTLLV	GDSLCEFTVKERNIEFKAVLAYEPKK
Class I	Blr	41	MNRLIE	LTGWIVLVVSVILLGVASHI	DNYQPPEQSASVQHK
Class I	YshB	36	MLESIINLVSSGAV	DSHTPQTAVAAVLCAAMIGLF	S
Class II	YoaJ	24	MKKT	TIIMMGVAIIVVLGTELGW	W
Class II	YncL	31	MNVSSR	TVVLINFFAAVGLFTLISMRFGW	FI
Class II	YthA	41	MIKNFIFDN	LIILAVPFMIKTSLKTNLIFFFL	CVFVPHMAS
Class II	YoaK	32	MRIGII	FPVVIFITAVVFLAWFFIGGYAA	PGA
Class II	HokC	50	MKQHKA	MIVALIVICITAVVAALV	TRKDLCEVHIRTGQTEVAVFTAYESE
Class II	HokD	51	MKQQKA	MLIALIVICLTVIVTALVT	RKDLCEVRIRTGQTEVAVFTAYEPEE
Class II	MgrB	47	MKKFRW	VVLVVVVLACLLLWAQVF	NMMCDQDVQFFSGICAINQFIPW
Class II	CydX	37	M	WYFAWILGTLLACSFGVITALAL	EHVESGKAGQEDI

FIG S6 The amino acid sequences of RseP-cleavable SMPs. The amino acid sequences of RseP-cleavable SMPs and RseA148 (DegS-cleaved form of RseA) are shown. The transmembrane regions (shown in red) of the SMPs were predicted using TMHMM (32) or TOPCONS (33) (see Table S1). Class I and Class II SMPs are shaded in blue and orange, respectively.
