# Supplementary material for: S2P intramembrane protease RseP degrades small membrane proteins and suppresses the cytotoxicity of intrinsic toxin HokB
Source: mBio. 2023 Jul 6;14(4):e01086-23. doi: 10.1128/mbio.01086-23 (PMC10470546; doi:10.1128/mbio.01086-23)
Supplement: Supplementary data — Supplemental materials and methods [file mbio.01086-23-s0007.docx]

**Supplemental material for**

S2P intramembrane protease RseP degrades small membrane proteins and suppresses the cytotoxicity of intrinsic toxin HokB

Tatsuhiko Yokoyama^a^, Yutaro Yamagata^a^, Saisei Honna^a^, Shinya Mizuno^a^, Shizuka Katagiri^b^, Rika Oi^b^, Terukazu Nogi^b^, Yohei Hizukuri^a, 1^, and Yoshinori Akiyama^a, 1^

^a^Institute for Life and Medical Sciences, Kyoto University, 53 Shogoinkawahara-cho, Sakyo-ku, Kyoto 606-8507, Japan; ^b^Graduate School of Medical Life Science, Yokohama City University, 1-7-29 Suehiro-cho, Tsurumi-ku, Yokohama 230-0045, Japan

^1^To whom correspondence may be addressed. Email: yhizukur@infront.kyoto-u.ac.jp (Y.H.); yakiyama@infront.kyoto-u.ac.jp (Y.A.).

**Supplemental materials and methods**

Media. L broth (10 g/L Bacto Tryptone, 5 g/L yeast extract, and 5 g/L NaCl; pH adjusted to 7.2 with NaOH) was used to grow *E. coli* cells. Ampicillin (50 μg/mL), chloramphenicol (20 μg/mL) and/or spectinomycin (50 μg/mL) were added to the media for selecting transformants and for growing plasmid-harboring cells. Bacterial growth was monitored using mini photo 518R (660 nm; TAITEC).

Antibodies. Mouse monoclonal anti-FLAG M2 antibody (MilliporeSigma), anti-Myc antibody (Santa Cruz), Rabbit polyclonal anti-RseP (1), anti-SecB (2), anti-MBP (3) antibodies were used for immunoblotting.

Construction of strains. YK175 (Δ*hokB-175*::*kan*) was constructed by deleting the *hokB-sokB-mokB* region of BW25113 using the one-step method as described by Datsenko and Wanner (2000) (4) with pKD13, pKD46, and the primers P1 and P2. The *sokB* and *mokB* genes overlap with the *hokB* gene and regulate the expression of *hokB* (5)*.* For the construction of the Δ*hokB-175*::*kan* mutation, the primers P1 and P2 were designed to delete the region from -146 to +211 (the first nucleotide in the initiation codon of the *hokB* gene was set to +1), which contains the entire DNA sequences of the *hokB*, *sokB* and *mokB* genes (*hokB-locus*). YK202 was constructed by transferring the Δ*hokB-175*::*kan* marker from YK175 to YK167 by P1 transduction followed by deletion of the *kan* cassette using pCP20. YK225 was constructed by transferring the Δ*rseP*::*kan* marker from KK377 to YK202 by P1 transduction. KA304 was constructed by transferring the *clpP*::*cat* marker from MC4100 *clpP*::*cat* (provided by M. Kitagawa) to AD2543.

Construction of plasmids. pYH522 was constructed as follows. A fragment containing the entire *flm* locus (*flmA*/*flmB*/*flmC*) with EcoRI and SacI sites at the 5’ end and a BamHI site at the 3’ end was amplified from the F' plasmid of the *E. coli* strain AD16 by colony PCR with primers, P101/P102. After digestion with EcoRI and BamHI, the fragment was cloned into the same sites of pTWV228. pEB82 (pTWV228 HA-MBP-YqfG) was constructed as follows. A fragment containing the entire *yqfG* ORF with a SalI site at the 5’ end and a PstI site at the 3’ end was amplified from the *E. coli* chromosome by colony PCR with primers P3/P4. After digestion with SalI and PstI, the fragment was cloned into the same sites of pSTD835. A series of plasmids encoding HA-MBP-SMP (i.e., pEB63~pEB81, pEB83~pEB87, pEB90~pEB103, pEB105, pYK291, pYK58, and pYK4) were constructed essentially as described for pEB82 except pYK58 and pKY4, for which the *tisB* gene and the *flmA* gene without their own start codon were amplified rom the *E.coli* chromosome and pYH522, respectively, and cloned into pSTD835. pYH341 (HA-msfGFP-RseA(2-216)) was constructed as follows. First, the KpnI site of pKK55 was converted to an NheI site by site-directed mutagenesis using primers, P103/P104, and then the resulting plasmid was digested with NheI and ligated with an NheI-msfGFP-NheI cassette from pYH337 (6). pYH354 (HA-msfGFP-RseA(97-216)) was constructed as follows. A fragment containing the HA-msfGFP sequence with a SacI site at the 5' end and a BamHI site at the 3' end, respectively, was amplified from pYH341 with primers, P105/P106. After digestion with SacI and BamHI, the fragment was cloned into the same sites of pSTD795. pYH365 was constructed as follows. First, the BamHI site of pYH354 was converted to a SalI site by site-directed mutagenesis with primers, P107/P108, and then an EcoRI/PstI fragment of the resulting plasmid was cloned into the same sites of pTWV228. pYH367, pYH368, pYH369, pYH370, pYH371, and pYH372 were constructed by cloning the SalI/PstI fragment of pEB70, pEB72, pEB82, pEB87, pEB101, and pEB74, respectively, into the same sites of pYH365. pYY17 was a derivative of pKK55 and constructed by the following 3 step mutagenesis; (i) site-directed mutagenesis using pKK55 (template) and primers P5/P6, (ii) replacement of the SalI-HindIII fragment with that of pYH367, and (iii) site-directed mutagenesis using primers P7/P8. pYY21 was constructed by cloning a SalI/HindIII fragment of pYH370 into the same sites of pYY17. pYY83 was constructed as follows. A fragment containing the 3xFLAG sequence with a SacI site at the 5’ end and a SalI site at the 3’ end was amplified from a laboratory-stock plasmid encoding 3xFLAG sequence by PCR with primers P9/P10. After digestion with SacI and SalI, the 3xFLAG-encoding fragment was cloned into the same sites of pYY21. pYY94, pYY96, pYY98, pYY100, and pYY102 were constructed by cloning the SalI/HindIII fragment of pYH367, pYH368, pYH369, pYH371, and pYH372 into the same sites of pYY83, respectively. pYK78, pYK87, pYK91, pYK93, pYK94, pYK96, pYK295, and pYK297 were constructed by cloning the SalI/PstI fragment of pEB99, pEB76, pEB63, pEB64, pEB65, pEB71, pEB100, and pYK291 into the same sites of pYY83, respectively. pYK347 was constructed as follows. A fragment encoding the initiation methionine-less RseA148 with a SalI site at the 5’ end and an ochre codon plus a PstI site at the 3’ end was amplified from the *E. coli* chromosome by colony PCR with primers P11/P12. After digestion with SalI and PstI, the fragment was cloned into the same sites of pYY83. pYK99 was constructed by site-directed mutagenesis of pYK78 to delete the sequence corresponding to the 3xFLAG tag using primers P13/P14. pYK412 was constructed by cloning the EcoRI/HindII fragment of pYK99 into the same sites of pBAD18. To obtain pYY44, pYY46, pYY47, pYY48, pYY50, and pYY51, the NheI/SalI-digested vector fragment of pYH368, pYH369, pYH370, pYH371, pYH372, and pYH367 were ligated with the annealed oligonucleotides P15/P16. pNY1425 had essentially the same structure as pNY1452 except that it encoded the E23Q mutant version of RseP.

Production and purification of wild-type RseP and the E23Q mutant. RseP(WT) used for the *in vitro* cleavage assay was prepared essentially as reported previously (7). RseP(WT) was produced in *E. coli* C43(DE3) (Lucigen) transformed with the expression plasmid pNY1452. The *E. coli* cells were grown at 30 °C to an OD_600_ of 0.7–0.8 in 6 L of LB medium (10 g/L Bacto Tryptone, 5 g/L yeast extract and 10 g/L NaCl; without pH adjustment) supplemented with 50 μg/mL ampicillin. Subsequently, RseP expression was then induced with 0.1 mM IPTG, and incubated at 30 °C for another 4 h. Cells were harvested by centrifugation and lysed by sonication in buffer containing 10 mM Tris-HCl (pH 7.4) and 150 mM NaCl. After clarification by centrifugation at 40,000 × *g* for 45 min at 4 °C, the cell lysates were was further ultracentrifuged at 200,000 × *g* for 90 min at 4 °C. The precipitate was suspended again in 10 mM Tris-HCl (pH 7.4)–150 mM NaCl and was ultracentrifuged under the same conditions. Finally, the precipitate was collected as the membrane fraction and was suspended in 10 mM Tris-Cl (pH 7.4) –150 mM NaCl. Total protein in the membrane fraction was quantified by the bicinchoninic acid (BCA) assay using bovine serum albumin as a standard. The resuspended membrane fraction was diluted to adjust the protein concentration to 10 mg/mL.

RseP was solubilized by adding an equal volume of buffer containing 40 mM Tris-HCl (pH 8.5), 150 mM NaCl, and 2% *s*ucrose *m*onododecanoate (SM) to the suspension of the membrane fraction prepared as above. After incubation at 4 °C for 1 h, the mixture was ultracentrifuged at 210,000 ×g for 90 min. at 4 °C. The supernatant was collected as the solubilizate and was applied to NZ-1 antibody conjugated Sepharose resins (anti-PA tag). After collecting the flow-through fraction, the resins were washed with a buffer containing 10 mM Tris-HCl (pH 8.5), 150 mM NaCl, and 0.05% SM. RseP was then eluted from the resins with a buffer containing 10 mM Tris-HCl (pH 8.5), 150 mM NaCl, 0.05% SM, and 0.1 mg/mL PA14 peptide (EGGVAMPGAEDDVV). After cleavage of the C-terminal tag by overnight incubation with TEV protease at 20 °C, tag cleavage was confirmed by the band shift on SDS-PAGE. The reaction mixture was then applied to a Superdex 200 Increase 10/300 GL size-exclusion chromatography (SEC) column (Cytiva) to remove oligomeric RseP and aggregated TEV protease. The peak fraction containing monomeric RseP was collected and reapplied to the same SEC column to increase purity. After the second round of SEC, the purified monodisperse RseP sample was concentrated by ultrafiltration using Amicon Ultra-0.5 (Merck Millipore) with a molecular mass cut-off of 30 kDa. RseP(E23Q) was overproduced in *E. coli* C43(DE3) cells transformed with the expression plasmid pNY1425 and purified using the same protocol as that for the wild type.

Immunoblotting. Immunoblotting was carried out essentially as described previously (8). Briefly, proteins were separated by SDS-PAGE and electroblotted onto an Immobilon-P membrane filter (MilliporeSigma). When 15% Bis-Tris gel was used for SDS-PAGE, a transferred membrane filter was dried at 37 °C for 30-60 min and then hydrophilized with methanol. After blocking with BLOTTO, the filter was incubated with an appropriate antibody. For anti-RseP immunoblotting, anti-RseP antibodies were pre-incubated with whole-cell lysates of AD1840 (the Δ*rseA* Δ*rseP* Δ*degS* strain) at 4 °C for about 1 h to reduce a background. The filter was then washed and incubated with goat anti-mouse or anti-rabbit IgG conjugated with horseradish peroxidase (Bio-Rad). After the filter was washed, proteins that reacted with secondary antibodies were visualized using ECL or ECL Prime Western Blotting Detection Reagents (Cytiva) and Bioimage analyzer LAS4000mini (Cytiva) or LAS3000mini (Cytiva).

*In vitro* cleavage assay. Model substrates were *in vitro* synthesized by using PURE*frex*^®^ 1.0 reconstituted cell-free protein synthesis kit (Gene Frontier) (9, 10). To prepare templates for *in vitro* synthesis, DNA fragments encoding HA-Met_6_-SMP were amplified by two-step PCR reactions. For HA-Met_6_-YncL, -YqfG, -YkgR, -HokE, -YoaJ, and -Blr, the first PCR reactions were carried out with the primers P17/P18, P17/P19, P17/P20, P17/P21, P17/P22, and P17/P23, respectively, and the plasmids pYY44, pYY46, pYY47, pYY48, pYY50, and pYY51, respectively, as templates. The second PCR reactions were carried out using the first PCR products templates using primers P40/P18, P40/P19, P40/P20, P40/P21, P40/P22, and P40/P23, respectively. For HA-Met_6_-HokB, -YthA, -YoaK, -HokC, -HokD, -MgrB, -CydX, and -YshB, the first PCR reactions were carried with the primers P24/P25, P26/P27, P28/P29, P30/P31, P32/P33, P34/P35, P36/P37, and P38/P39, respectively, and the plasmids pEB99, pEB65, pEB63, pEB100, pYK291, pEB76, pEB71, and pEB64, respectively, as templates. The second PCR reactions were carried out using the first PCR products as templates using primers P40/P25, P40/P27, P40/P29, P40/P31, P40/P33, P40/P35, P40/P37, and P40/P39, respectively.

The DNA templates were added to the PURE*frex*^®^ 1.0 reaction reagent in the presence of 0.02% *n*-dodecyl-*β*-D-maltoside (DDM), 1/40 volume of recombinant RNase inhibitor (Takara), and 37 MBq/L [^35^S]-labeled methionine (^35^S-Met), and incubated at 37 °C for 1 h. After terminating the reaction by addition of 0.15 g/L chloramphenicol, 2.25 μL of the reaction solutions containing the synthesized [^35^S]-labeled substrates were mixed with 1 μL of purified RseP [20 ng/μL (400 nM) for Fig 5*A*, or 250 ng/μL (5 μM) for Fig. 5*B*, *C*, *D,* and *E*] or buffer for purified RseP, 0.5 μL of 200 mM *β-*mercaptoethanol, 4.2 μL of dialysis buffer (10 mM Tris-HCl (pH 8.1), 300 mM KCl, 5% (w/v) glycerol, 0.02% DDM), 0.25 μL of 4 M NaCl, 1 μL of RseP buffer (500 mM Tris-HCl (pH 8.1), 50 μΜ zinc-acetate, 25% (w/v) glycerol, 0.2 % DDM), 0.1 μL of Protease Inhibitor Cocktail (EDTA free) (Nacali Tesque), 0.1 μL of 100 μM phenylmethylsulfonyl fluoride (PMSF), 0.1 μL of 100 μM Pefabloc® SC (Merck), and 0.5 μL of 0 or 100 mM *1,10*-phenanthroline dissolved in DMSO, and incubated at 37 °C for the indicated periods. A portion of the samples were withdrawn at intervals and mixed with the same volume of 2xSDS sample buffer (pH, 8.5). After vigorous shaking for 10 min at room temperature, followed by heating at 99 °C for 10 min, the proteins were separated by SDS-PAGE using a 15% Bis-Tris gel and MES SDS running buffer (8) and visualized using a phosphor imager BAS5000 (Cytiva).

**References for supplemental materials and methods**

1. Hizukuri Y, Akiyama Y. 2012. PDZ domains of RseP are not essential for sequential cleavage of RseA or stress-induced σ^E^ activation *in vivo*. Mol Microbiol 86:1232–1245.

2. Miyake T, Hizukuri Y, Akiyama Y. 2020. Involvement of a Membrane-bound amphiphilic helix in substrate discrimination and binding by an *Escherichia coli* S2P peptidase RseP. Front Microbiol 11:607381.

3. Akiyama Y, Kanehara K, Ito K. 2004. RseP (YaeL), an *Escherichia coli* RIP protease, cleaves transmembrane sequences. EMBO J 23:4434–4442.

4. Datsenko KA, Wanner BL. 2000. One-step inactivation of chromosomal genes in *Escherichia coli* K-12 using PCR products. Proc Natl Acad Sci U S A 97:6640–6645.

5. Pedersen K, Gerdes K. 1999. Multiple *hok* genes on the chromosome of *Escherichia coli*. Mol Microbiol 32:1090–1102.

6. Yoshitani K, Hizukuri Y, Akiyama Y. 2019. An *in vivo* protease activity assay for investigating the functions of the *Escherichia coli* membrane protease HtpX. FEBS Lett 593:842–851.

7. Imaizumi Y, Takanuki K, Miyake T, Takemoto M, Hirata K, Hirose M, Oi R, Kobayashi T, Miyoshi K, Aruga R, Yokoyama T, Katagiri S, Matsuura H, Iwasaki K, Kato T, Kaneko MK, Kato Y, Tajiri M, Akashi S, Nureki O, Hizukuri Y, Akiyama Y, Nogi T. 2022. Mechanistic insights into intramembrane proteolysis by *E. coli* site-2 protease homolog RseP. Sci Adv 8:eabp9011.

8. Yokoyama T, Niinae T, Tsumagari K, Imami K, Ishihama Y, Hizukuri Y, Akiyama Y. 2021. The *Escherichia coli* S2P intramembrane protease RseP regulates ferric citrate uptake by cleaving the sigma factor regulator FecR. J Biol Chem 296:100673.

9. Shimizu Y, Kanamori T, Ueda T. 2005. Protein synthesis by pure translation systems. Methods 36:299–304.

10. Shimizu Y, Inoue A, Tomari Y, Suzuki T, Yokogawa T, Nishikawa K, Ueda T. 2001. Cell-free translation reconstituted with purified components. Nat Biotechnol 19:751–755.
