# Supplementary material for: S2P intramembrane protease RseP degrades small membrane proteins and suppresses the cytotoxicity of intrinsic toxin HokB
Source: mBio. 2023 Jul 6;14(4):e01086-23. doi: 10.1128/mbio.01086-23 (PMC10470546; doi:10.1128/mbio.01086-23)
Supplement: Table S2 — Strains, plasmids, and oligonucleotides. [file mbio.01086-23-s0009.docx]

Table S2. The strains, plasmids, and oligonucleotides used in this study.

| ***E. coli* strains** | | |
| --- | --- | --- |
| Name | Genotype | Reference |
| MC4100 | F^-^, *araD139* Δ*(argF-lac)U169 rpsL150 relA1 flbB5301 deoC1 ptsF25 rbsR* | (1) |
| CU141 | MC4100 /F’ *lacI*^q^ *Z*^+^ *Y*^+^ | (2) |
| KK377 | CU141, Δ*rseA*::*cat* Δ*rseP*::*kan* | (3) |
| HM1742 | CU141, *ara^+^* | (4) |
| YK167 | HM1742, Δ*rseA* | (5) |
| YK191 | HM1742, Δ*rseA* Δ*rseP*∷*kan* | (5) |
| YK202 | HM1742, Δ*rseA* Δ*hokB-175* [Δ(*hokB-locus*)] | This study |
| YK225 | HM1742, Δ*rseA* Δ*hokB-175* Δ*rseP*::*kan* | This study |
| BW25113 | F^-^, *rrnB* Δ*lacZ4787* *hsdR514* Δ(*araBAD*)*567* Δ(*rhaBAD*)*568* *rph-1* | (6) |
| YK175 | BW25113, Δ*hokB-175*∷*kan* | This study |
| JW2556 | BW25113, Δ*rseA*∷*kan,* KEIO collection | (7) |
| AD16 | Δ*pro-lac thi* /F’ *lacI*^q^ *Z*ΔM15 *Y*^+^ *pro*^+^ | (8) |
| AD2543 | AD16, Δ*rseA* | (9) |
| KA304 | AD16, Δ*rseA* Δ*clpP*::*cat* | This study |
| KA306 | AD16, Δ*rseA* Δ*rseP*::*kan* Δ*clpP*::*cat* | (10) |
| AD1840 | AD16, Δ*rseA*::*cat* Δ*rseP*::*kan* Δ*degS*::*tet* | (11) |
| C43(DE3) | F^-^, *ompT* *hsdS*_B_(r_B_^-^ m_B_^-^) *gal dcm* (DE3) | Lucigen |

| **Plasmids** | | | |
| --- | --- | --- | --- |
| Name | Vector | ﻿Encoded proteins or descriptions | Reference |
| pTWV228 |  | pBR322-based vector; P_lac_, Amp^R^ | Takara Bio |
| pUC118 |  | pBR322-based vector; P_lac_, Amp^R^ | Takara Bio |
| pSTD689 |  | pACYC184-based vector; P_lac_, Spc^R^ | (12) |
| pBAD18 |  | pBR322-based vector; P_araBAD_, Amp^R^ | (13) |
| pKD13 |  | Template for the PCR of cassette construction | (6) |
| pKD46 |  | λ-Red recombinase system | (6) |
| pCP20 |  | ﻿FLP recombinase | (14) |
| pYH9 | pSTD689 | RseP-His_6_-Myc | (15) |
| pYH13 | pSTD689 | RseP(E23Q)-His_6_-Myc | (15) |
| pSTD835 | pTWV228 | HA-MBP-RseA(LY1)140 | (3) |
| pYH20 | pTWV228 | HA-MBP-RseA(LY1)148 | (15) |
| pKK55 | pTWV228 | HA-RseA | (3) |
| pEB82 | pTWV228 | HA-MBP-YqfG | This study |
| pEB74 | pTWV228 | HA-MBP-YoaJ | This study |
| pEB63 | pTWV228 | HA-MBP-YoaK | This study |
| pEB64 | pTWV228 | HA-MBP-YshB | This study |
| pEB65 | pTWV228 | HA-MBP-YthA | This study |
| pEB66 | pTWV228 | HA-MBP-YmiA | This study |
| pEB67 | pTWV228 | HA-MBP-YceO | This study |
| pEB68 | pTWV228 | HA-MBP-YohO | This study |
| pEB69 | pTWV228 | HA-MBP-AppX | This study |
| pEB70 | pTWV228 | HA-MBP-Blr | This study |
| pEB71 | pTWV228 | HA-MBP-CydX | This study |
| pEB72 | pTWV228 | HA-MBP-YncL | This study |
| pEB73 | pTWV228 | HA-MBP-YpdK | This study |
| pEB75 | pTWV228 | HA-MBP-YniD | This study |
| pEB76 | pTWV228 | HA-MBP-MgrB | This study |
| pEB77 | pTWV228 | HA-MBP-MgtS | This study |
| pEB78 | pTWV228 | HA-MBP-CydH | This study |
| pEB79 | pTWV228 | HA-MBP-YhoP | This study |
| pEB80 | pTWV228 | HA-MBP-YdgU | This study |
| pEB81 | pTWV228 | HA-MBP-AcrZ | This study |
| pEB83 | pTWV228 | HA-MBP-YoaI | This study |
| pEB84 | pTWV228 | HA-MBP-KdpF | This study |
| pEB85 | pTWV228 | HA-MBP-DinQ | This study |
| pEB86 | pTWV228 | HA-MBP-AzuC | This study |
| pEB87 | pTWV228 | HA-MBP-YkgR | This study |
| pEB90 | pTWV228 | HA-MBP-YaaY | This study |
| pEB91 | pTWV228 | HA-MBP-FtsL | This study |
| pEB92 | pTWV228 | HA-MBP-YbfA | This study |
| pEB93 | pTWV228 | HA-MBP-SixA | This study |
| pEB94 | pTWV228 | HA-MBP-ElaB | This study |
| pEB95 | pTWV228 | HA-MBP-YgaM | This study |
| pEB96 | pTWV228 | HA-MBP-GspI | This study |
| pEB97 | pTWV228 | HA-MBP-YhfU | This study |
| pEB98 | pTWV228 | HA-MBP-HokA | This study |
| pEB99 | pTWV228 | HA-MBP-HokB | This study |
| pEB100 | pTWV228 | HA-MBP-HokC | This study |
| pEB101 | pTWV228 | HA-MBP-HokE | This study |
| pEB102 | pTWV228 | HA-MBP-LdrA | This study |
| pEB103 | pTWV228 | HA-MBP-LdrD | This study |
| pEB105 | pTWV228 | HA-MBP-YqaE | This study |
| pYK291 | pTWV228 | HA-MBP-HokD | This study |
| pYK58 | pTWV228 | HA-MBP-TisB | This study |
| pYK4 | pTWV228 | HA-MBP-FlmA | This study |
| pYK347 | pTWV228 | 3xFLAG-RseA148 | This study |
| pYY83 | pTWV228 | 3xFLAG-YkgR | This study |
| pYY94 | pTWV228 | 3xFLAG-Blr | This study |
| pYY96 | pTWV228 | 3xFLAG-YncL | This study |
| pYY98 | pTWV228 | 3xFLAG-YqfG | This study |
| pYY100 | pTWV228 | 3xFLAG-HokE | This study |
| pYK78 | pTWV228 | 3xFLAG-HokB | This study |
| pYK87 | pTWV228 | 3xFLAG-MgrB | This study |
| pYK91 | pTWV228 | 3xFLAG-YoaK | This study |
| pYK93 | pTWV228 | 3xFLAG-YshB | This study |
| pYK94 | pTWV228 | 3xFLAG-YthA | This study |
| pYK96 | pTWV228 | 3xFLAG-CydX | This study |
| pYK295 | pTWV228 | 3xFLAG-HokC | This study |
| pYK297 | pTWV228 | 3xFLAG-HokD | This study |
| pYY102 | pTWV228 | 3xFLAG-YoaJ | This study |
| pYK99 | pTWV228 | HokB | This study |
| pYK412 | pBAD18 | HokB | This study |
| pYY44 | pTWV228 | HA-Met_6_-YncL | This study |
| pYY46 | pTWV228 | HA-Met_6_-YqfG | This study |
| pYY47 | pTWV228 | HA-Met_6_-YkgR | This study |
| pYY48 | pTWV228 | HA-Met_6_-HokE | This study |
| pYY50 | pTWV228 | HA-Met_6_-YoaJ | This study |
| pYY51 | pTWV228 | HA-Met_6_-Blr | This study |
| pYY17 | pTWV228 | HA-Blr | This study |
| pYY21 | pTWV228 | HA-YkgR | This study |
| pYH522 | pTWV228 | *flmABC* *locus* | This study |
| pYH341 | pTWV228 | HA-msfGFP-RseA(2-216) | This study |
| pYH337 | pUC118 | NheI-msfGFP-NheI cassette | (16) |
| pSTD795 | pTWV228 | HA-MBP-RseA(97-216) | (3) |
| pYH354 | pTWV228 | HA-msfGFP-RseA(97-216) | This study |
| pYH365 | pTWV228 | HA-msfGFP-RseA(TM) | This study |
| pYH367 | pTWV228 | HA-msfGFP-Blr | This study |
| pYH368 | pTWV228 | HA-msfGFP-YncL | This study |
| pYH369 | pTWV228 | HA-msfGFP-YqfG | This study |
| pYH370 | pTWV228 | HA-msfGFP-YkgR | This study |
| pYH371 | pTWV228 | HA-msfGFP-HokE | This study |
| pYH372 | pTWV228 | HA-msfGFP-YoaJ | This study |
| pNY1452 | pUC118 | RseP-TEV-His_8_-Myc-PA | (9) |
| pNY1425 | pUC118 | RseP(E23Q)-TEV-His_8_-Myc-PA | This study |

| **Oligonucleotides** | | |
| --- | --- | --- |
| No. | Name | Sequence |
| P1 | hokB_loci_disrupt(+) | GATTATTTTCGTCCCTAAGGAAACCTCATAGGATGCCTGCGTGTAGGCTGGAGCTGCTTC |
| P2 | hokB_loci_disrupt(-) | GTGCATCGAAAAAAACATGCACTAACTGAAGTGTAAAAGCATATGAATATCCTCCTTAG |
| P3 | yqfG-5-Sal1 | ﻿ACGCGTCGACATGAATTTTTTAATGCGCGC |
| P4 | yqfG-3-Pst1 | AACTGCAGTTAAAAAAGTAGCATCATAT |
| P5 | 5723ha-rseA SalI(+) | ATTATGCCTCGTCGACTCAGAAAGAACAACTTTC |
| P6 | 5723ha-rseA SalI(-) | TTCTTTCTGAGTCGACGAGGCATAATCTGGAAC |
| P7 | 5805pYY9G(+) | ATTATGCCTCGGTCGACATGAATCGTC |
| P8 | 5805pYY9G(-) | CATGTCGACCGAGGCATAATCTGGAAC |
| P9 | 6728YYSacI-3xFlag(plus) | CCCGAGCTCGGTACAAGGAGGAAGAGCAAATGGACTACAAAGAC |
| P10 | 6728YY3xFlag-SalI(-) | CCCCCGTCGACCTTGTCGTCATCGTCTTTG |
| P11 | 210629_Sal1-rseA(p) | ACGCGTCGACCAGAAAGAACAACTTTCCGC |
| P12 | 210629_rseA-Pst1(m) | AACTGCAGTTATACCGGGCTGGCTTTACC |
| P13 | 190417_tag-free_hokB(p) | GGTACAAGGAGGAAGAGCAAATGAAGCACAACCCTCTGG |
| P14 | 190417_tag-free_hokB(m) | CCAGAGGGTTGTGCTTCATTTGCTCTTCCTCCTTGTACC |
| P15 | NheI-met6-SalI(+) | CTAGCATGATGATGATGATGATGG |
| P16 | NheI-met6-SalI(-) | TCGACCATCATCATCATCATCATG |
| P17 | 5908RBS-HA(+) | AAGGAGATATACCAATGTATCCATATGATGTTCCAG |
| P18 | 6510YncL_short(-) | GCCTGCAGTCAAATAAACCAGCCAAATCTC |
| P19 | YqfG_short(-) | CATGCCTGCAGTTAAAAAAGTAGCATC |
| P20 | YkgR_short(-) | GCCTGCAGTTAATAGAAATGGAG |
| P21 | YY6X11HokE_short(-) | CATGCCTGCAGCTACTTCTTCGGTTCG |
| P22 | YY6X11YoaJ_short(-) | CATGCCTGCAGTTACCACCATCCCAGCTC |
| P23 | YY6X11Blr_short(-) | CATGCCTGCAGTTACTTGTGTTGTACCG |
| P24 | 220607_PURE_HA-Met6-HokB(p) | AAGGAGATATACCAATGTATCCATATGATGTTCCAGATTATGCCTCGGCTAGCATGATGATGATGATGATGGTCGACATGAAGCACAACCCTCTGGTG |
| P25 | 210629_PURE_F-Met6-HokB(m) | GGATTAGTTATTCATTACCTGGACGTGCAGGC |
| P26 | 220620_PURE_HA-Met6-YthA(p) | AAGGAGATATACCAATGTATCCATATGATGTTCCAGATTATGCCTCGGCTAGCATGATGATGATGATGATGGTCGACATGATCAAGAATTTTATCTTC |
| P27 | 220207_PURE_Met6-YthA(m) | GGATTAGTTATTCATTATGACGCCATATGGGG |
| P28 | 220620_PURE_HA-Met6-YoaK(p) | AAGGAGATATACCAATGTATCCATATGATGTTCCAGATTATGCCTCGGCTAGCATGATGATGATGATGATGGTCGACATGCGAATCGGTATTATTTTTC |
| P29 | 220207_PURE_Met6-YoaK(m) | GGATTAGTTATTCATTATGCTCCCGGGGCAG |
| P30 | 220620_PURE_HA-Met6-HokC(p) | AAGGAGATATACCAATGTATCCATATGATGTTCCAGATTATGCCTCGGCTAGCATGATGATGATGATGATGGTCGACATGAAGCAGCATAAGGCG |
| P31 | 220207_PURE_Met6-HokC(m) | GGATTAGTTATTCATTACTCGGATTCGTAAGCC |
| P32 | 220620_PURE_HA-Met6-HokD(p) | AAGGAGATATACCAATGTATCCATATGATGTTCCAGATTATGCCTCGGCTAGCATGATGATGATGATGATGGTCGACATGAAGCAGCAAAAGGCGA |
| P33 | 220207_PURE_Met6-HokD(m) | GGATTAGTTATTCATTACTCCTCAGGTTCGTAAG |
| P34 | 220620_PURE_HA-Met6-MgrB(p) | AAGGAGATATACCAATGTATCCATATGATGTTCCAGATTATGCCTCGGCTAGCATGATGATGATGATGATGGTCGACATGAAAAAGTTTCGATGGGTC |
| P35 | 220207_PURE_Met6-MgrB(m) | GGATTAGTTATTCATCACCACGGGATAAACTG |
| P36 | 220620_PURE_HA-Met6-CydX(p) | AAGGAGATATACCAATGTATCCATATGATGTTCCAGATTATGCCTCGGCTAGCATGATGATGATGATGATGGTCGACATGTGGTATTTCGCATGGAT |
| P37 | 220207_PURE_Met6-CydX(m) | GGATTAGTTATTCATCAGATGTCTTCTTGACCG |
| P38 | 220620_PURE_HA-Met6-YshB(p) | AAGGAGATATACCAATGTATCCATATGATGTTCCAGATTATGCCTCGGCTAGCATGATGATGATGATGATGGTCGACATGCTGGAATCAATAATTAATCT |
| P39 | 220207_PURE_Met6-YshB(m) | GGATTAGTTATTCATCAGCTAAACAGCCCAATC |
| P40 | T7 PRO-SD | GAAATTAATACCGACTCACTATAGGGAGACCACAACGGTTTCCCTCTAGAAATAATTTTGTTTAACTTTAAGAAGGAGATATACCA |
| P101 | 170619EcoSac-flm(p) | GGAATTCGAGCTCCGTAAACAGCCTGAATGAGCGGG |
| P102 | 170619flm-Bam(m) | CGCGGATCCGGGGCCACCGGCGAACCAGC |
| P103 | 140909HA-Nhe-RseA(p) | GCCTCGGCTAGCCAGAAAGAACAACTTTC |
| P104 | 140909HA-Nhe-RseA(m) | GTTCTTTCTGGCTAGCCGAGGCATAATCTG |
| P105 | RVN | TGTGGAATTGTGAGCGG |
| P106 | 141110GFPRseA_Nhe_to_Bam(m) | CTTTCTGGGATCCCTTGTACAGCTCGTCC |
| P107 | 150119GFP-RseATM_Bam_to_Sal(p) | GCTGTACAAGGTCGACTGGGCGGCACAGC |
| P108 | 150119GFP-RseATM_Bam_to_Sal(m) | GCCGCCCAGTCGACCTTGTACAGCTCGTCC |

**References for Table S2**

1. Silhavy T, Berman M, Enquist L. 1984. Experiments with Gene Fusions. Cold Spring Harbor Laboratory Press., Cold Spring Harbor, NewYork.

2. Akiyama Y, Ogura T, Ito K. 1994. Involvement of FtsH in protein assembly into and through the membrane. I. Mutations that reduce retention efficiency of a cytoplasmic reporter. J Biol Chem 269:5218–5224.

3. Akiyama Y, Kanehara K, Ito K. 2004. RseP (YaeL), an *Escherichia coli* RIP protease, cleaves transmembrane sequences. EMBO J 23:4434–4442.

4. Mori H, Ito K. 2006. The long *α*-helix of SecA is important for the ATPase coupling of translocation. J Biol Chem 281:36249–36256.

5. Yokoyama T, Niinae T, Tsumagari K, Imami K, Ishihama Y, Hizukuri Y, Akiyama Y. 2021. The *Escherichia coli* S2P intramembrane protease RseP regulates ferric citrate uptake by cleaving the sigma factor regulator FecR. J Biol Chem 296:100673.

6. Datsenko KA, Wanner BL. 2000. One-step inactivation of chromosomal genes in *Escherichia coli* K-12 using PCR products. Proc Natl Acad Sci U S A 97:6640–6645.

7. Baba T, Ara T, Hasegawa M, Takai Y, Okumura Y, Baba M, Datsenko KA, Tomita M, Wanner BL, Mori H. 2006. Construction of *Escherichia coli* K-12 in-frame, single-gene knockout mutants: the Keio collection. Mol Syst Biol 2:2006.0008.

8. Kihara A, Akiyama Y, Ito K. 1995. FtsH is required for proteolytic elimination of uncomplexed forms of SecY, an essential protein translocase subunit. Proc Natl Acad Sci U S A 92:4532–4536.

9. Imaizumi Y, Takanuki K, Miyake T, Takemoto M, Hirata K, Hirose M, Oi R, Kobayashi T, Miyoshi K, Aruga R, Yokoyama T, Katagiri S, Matsuura H, Iwasaki K, Kato T, Kaneko MK, Kato Y, Tajiri M, Akashi S, Nureki O, Hizukuri Y, Akiyama Y, Nogi T. 2022. Mechanistic insights into intramembrane proteolysis by *E. coli* site-2 protease homolog RseP. Sci Adv 8:eabp9011.

10. Akiyama K, Mizuno S, Hizukuri Y, Mori H, Nogi T, Akiyama Y. 2015. Roles of the membrane-reentrant β-hairpin-like loop of RseP protease in selective substrate cleavage. eLife 4:e08928.

11. Kanehara K, Ito K, Akiyama Y. 2002. YaeL (EcfE) activates the σ^E^ pathway of stress response through a site-2 cleavage of anti-σ^E^, RseA. Genes Dev 16:2147–2155.

12. Kanehara K, Ito K, Akiyama Y. 2003. YaeL proteolysis of RseA is controlled by the PDZ domain of YaeL and a Gln-rich region of RseA. EMBO J 22:6389–6398.

13. Guzman LM, Belin D, Carson MJ, Beckwith J. 1995. Tight regulation, modulation, and high-level expression by vectors containing the arabinose P_BAD_ promoter. J Bacteriol 177:4121–4130.

14. Cherepanov PP, Wackernagel W. 1995. Gene disruption in *Escherichia coli*: Tc^R^ and Km^R^ cassettes with the option of Flp-catalyzed excision of the antibiotic-resistance determinant. Gene 158:9–14.

15. Hizukuri Y, Akiyama Y. 2012. PDZ domains of RseP are not essential for sequential cleavage of RseA or stress-induced σ^E^ activation *in vivo*. Mol Microbiol 86:1232–1245.

16. Yoshitani K, Hizukuri Y, Akiyama Y. 2019. An *in vivo* protease activity assay for investigating the functions of the *Escherichia coli* membrane protease HtpX. FEBS Lett 593:842–851.
